# Citrulline Inhibits *Clostridioides difficile* Infection With Anti-inflammatory Effects

**DOI:** 10.1016/j.jcmgh.2025.101474

**Published:** 2025-02-07

**Authors:** Ying Xie, Sophie Irwin, Becca Nelson, Mieke van Daelen, Lindsey Fontenot, Jonathan P. Jacobs, Monica Cappelletti, Hanping Feng, Yiling Li, Hon Wai Koon

**Affiliations:** 1 Vatche and Tamar Manoukian Division of Digestive Diseases, David Geffen School of Medicine at the University of California Los Angeles, Los Angeles, California; 2 Department of Gastroenterology and Endoscopy, The First Hospital of China Medical University, Shenyang City, Liaoning Province, China; 3 Goodman-Luskin Microbiome Center, David Geffen School of Medicine at UCLA, Los Angeles, California; 4 Division of Gastroenterology, Hepatology and Parenteral Nutrition, VA Greater Los Angeles Healthcare System, Los Angeles, California; 5 Department of Microbial Pathogenesis, School of Dentistry, University of Maryland, Baltimore, Maryland; 6 Immunogenetics Division, Department of Pathology and Laboratory Medicine, David Geffen School of Medicine at the University of California Los Angeles, Los Angeles, California

**Keywords:** Inflammation, Metabolite, Microbiome

## Abstract

**Background & Aims:**

*Clostridioides difficile* infection (CDI) causes colitis and diarrhea. *C. difficile* bacterium produces toxins A and B, which cause intestinal inflammation. A metabolomics analysis discovered fecal metabolites with anti-inflammatory effects in CDI. We aimed to identify an anti-CDI metabolite that can inhibit CDI-mediated colitis and prevent recurrence.

**Methods:**

Fresh human colonic tissues and primary human cells were used to determine metabolite effects. Humanized *C. difficile*-infected HuCD34-NCG mice and antibiotics-treated human gut microbiota-treated (ABX + HGM) hamsters were used to simulate the human environment.

**Results:**

High-throughput screening and fecal metabolomics analysis identified anti-inflammatory metabolites. Compared with other tested metabolites, citrulline preserved the mucosal integrity of toxin-exposed fresh human colonic tissues with reduced macrophage inflammatory protein 1 alpha (MIP-1a) and increased interleukin-10 (IL-10) expression. Oral citrulline treatment alleviated cecal inflammation in hamsters infected with *C. difficile* ribotype 027. This was accomplished by the augmented expression of cecal IL-10 and the diminished level of cecal MIP-1a. Citrulline and vancomycin synergistically prevented recurrence in the infected ABX + HGM hamsters. In C57BL/6J mice infected with *C. difficile* VPI10463, citrulline ameliorated colitis by reducing colonic Ccl3 mRNA expression. In immunologically humanized HuCD34-NCG mice infected with toxin B-expressing *C. difficile* ribotype 017, citrulline ameliorated colitis with increased human IL-10 expression in colonic macrophages. Citrulline suppressed MIP-1a secretion and GSK3a/b dephosphorylation in the toxin A-exposed human colonic epithelial cells and promoted IL-10 expression in toxin B-exposed human macrophages and heat shock protein 27 phosphorylation.

**Conclusion:**

Citrulline exerts anti-inflammatory effects in the intestines against *C. difficile* toxins and inhibits CDI recurrence in mice and hamsters.

## Graphical abstract

**Figure:**
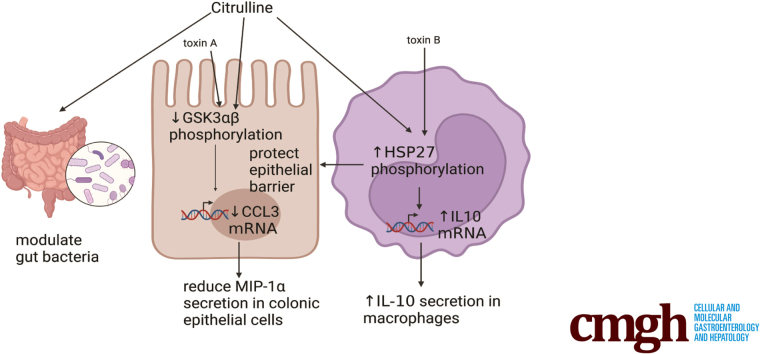

**Summary**

Citrulline inhibits *Clostridioides difficile* infection with reduced MIP-1a expression in toxin A-exposed intestinal epithelial cells, increased interleukin-10 expression in toxin B-exposed macrophages, and possibly modulation of gut microbiota.

*Clostridioides difficile* infection (CDI) is a debilitating nosocomial disease that affects many patients with extended antibiotic treatment. *C. difficile* bacteria produce toxins A and B, which cause colitis, diarrhea, and abdominal pain. Standard antibiotic (vancomycin) treatment can treat the primary infection but is associated with a high recurrence rate.^1^ About 20% of patients with CDI experience a recurrence ≥14 days after their first CDI.^2^

Fidaxomicin is not inferior to vancomycin in efficacy, with a lower recurrence rate than vancomycin.^3^ However, fidaxomicin therapy is expensive.^4^ For patients with metronidazole-resistant CDI, ritelimin can be beneficial in >65% of cases.^5^ Anti-toxin B neutralizing antibody bezlotoxumab may prevent recurrent CDI but with a modest, sustained cure rate (64%).^6^ Fecal microbiota transplantation (FMT) conferred a 90% successful cure rate, but it does not have approval from the United States Food and Drug Administration (FDA).^7^, ^8^, ^9^ Recently, REBYOTA and VOWST, live microbiota therapeutics, were approved to reduce CDI recurrence. Some patients are unresponsive to currently available therapies and eventually require surgery, emphasizing the continued need for novel therapeutic approaches.

Microbiota and host can affect the intestinal chemical environment, influencing disease activity. The involved chemicals can include host-derived, microbial, and dietary metabolites.

Metabolomic analysis of fecal samples from *C. difficile*-infected mice showed reduced fecal levels of citrulline, retinol, and ursodeoxycholic acid.^10^ These metabolites possess anti-inflammatory effects against toxin A in mouse macrophages.^10^ Oral treatments of these metabolites improved survival and prevented recurrence in the infected mice.^10^

These findings paved the way for a broader investigation to identify anti-inflammatory and antibacterial metabolites through multidimensional high-throughput screening (HTS) and to characterize their effects using state-of-the-art, clinically relevant immunologically and microbiologically humanized models. We hypothesized that a metabolite could exhibit multifaceted therapeutic effects against primary infection and recurrence of CDI.

## Results

### The Intestine is the Source of CDI-dependent Cytokines

Our previous studies demonstrated that *C. difficile* toxin-exposed human intestinal explants and circulating monocytes express many cytokines.^11^^,^^12^ However, the differences in serum levels of major cytokines and chemokines between healthy donors and patients with CDI were less than 2-fold (Table 2A, Table 2B). Similarly, CDI does not significantly affect circulating levels of macrophage inflammatory protein 1 alpha (MIP-1α), interleukin (IL)-1β, and tumor necrosis factor alpha (TNFα) in mice and MIP-1α in hamsters.^11^^,^^12^ These findings suggested that the intestine should be the probable source of cytokine production in CDI.

**Table 1A:** Information on Fresh Human Colonic Tissues, Primary Macrophages, PBMCs, and Reagents

	Vendor	Catalog #	Purity	Batch
Antibiotics for CDI				
Colistin sulfate	MCE	HY-A0089	98%	16647
Vancomycin hydrochloride	Sigma	94747	91.60%	028M4029V
Kanamycin	Sigma	K1876	692μg/mg	BCBC5819V
Metronidazole	Sigma	M3761	100%	SLBG3633V
Gentamycin sulfate	Fisher	BP918-1	590μg/mg	180621
Clindamycin hydrochloride	Sigma	PHR1159	90%	LRAC5692
Streptomycin	Sigma	S6501	761IU/mg	SLBT4470
Antibiotics in gnotobiotic hamsters				
Ertapenem sodium salt	Research Products Internationa	50-997-777	97%	174369-185642
Neomycin sulfate hydrate	Alta Aesar	J61499	600μg/mg	Z20C038
Ampicillin sodium salt	Fisher Scientific	BP1760-25	845μg/mg	055M4755V
Cefoperazone	Research Products Internationa	C27685G	870 - 1015 μg/mg	E8GUG-WS
CDI germinants				
Sodium taurocholate	Sigma	86339	99%	BCCB0835
BHIS	BD	237200	N/A	5076656
Metabolite-CDI project				
DL-3-aminoisobutyric acid	Sigma	217794	98.50%	#0000068807
L-Citrulline	TCI	C0372	98%	G73KL-CH
Retinol acetate water soluble	Sigma	R0635	N/A	SLBS0459V
Ursodeoxycholic acid	Alfa Aesar	B20490	99%	R16D058
L-NMMA acetate	MCE	HY-18732A	98%	
Human HSP27	R&D Systems	1580-HS-050	95%	IWB0721111
Human MIP-1α	Peprotech	300-08	98%	#070935
Human MIP-1α antibody	R&D Systems	AB-270-NA	N/A	CH0411101
Human IL-10	Peprotech	200-10	98%	#110621
Human IL-10 antibody	R&D Systems	AB-217-NA	N/A	EU1221111
Murine MIP-1α	Peprotech	250-09	98%	#1008125
Murine IL-10	Peprotech	210-10	98%	#051453
Murine IL-10 antibody	R&D Systems	Ab-417-NA	N/A	BQ0719041

Note: Reagent information and baseline characteristics of the donors of fresh human colonic explants, primary human macrophages, and PBMCs.

CDI, *Clostridioides difficile* infection; IL, interleukin; L-NMMA, NG-monomethyl-L-arginine acetate; MIP-1α, macrophage inflammatory protein 1 alpha; PBMC, primary human peripheral blood mononuclear cell.

**Table 1B:** Baseline Characteristics

**Fresh human colonic tissues**	
Male sex	50%
Mean age (years)	66
Location:	
Ascending colon	25%
Transverse colon	25%
Descending	25%
Rectum	25%
n	12
**Human macrophages**	
Viability	92%
Mean age (years)	37
Male sex	40%
Caucasian	60%
Other ethnicity	40%
Mean body weight (kg)	87
Mean body height	172
Smoker	10%
A+ blood type	50%
B+ blood type	40%
O+ blood type	10%
**Primary human peripheral blood mononuclear cells**	
Viability	97%
Mean age (years)	33
Male sex	50%
Caucasian	50%
Other ethnicity	50%
Mean body weight (kg)	88
Mean body height (cm)	173
Smoker	50%
A+ blood type	50%
B+ blood type	50%

**Table 2A:** Serum Proteomics of Healthy Donors and Patients With CDI

Baseline characteristics of serum donors		
Serum samples for proteomics	Healthy	CDI
Age at collection, years (mean ± standard deviation)	46 ± 13	57 ± 17
Gender (% male)	40	40
N	10	10

Note: Major cytokines and chemokines in healthy donors and patients with CDI were determined. Baseline characteristics of serum donors are shown. The sera were pooled from 10 patients per group. T-tests were used to compare healthy and CDI groups. No statistically significant differences were found in all cytokines between the two groups.

CDI, *Clostridioides difficile* infection.

**Table 2B:** 

**Gene**	**Proteins**	%	%	fold
		**Healthy**	**CDI**	**CDI**
HSPB1	**HSP27**	100	36	-2.78
IL1A	**IL-1 alpha**	100	69	-1.45
IL1B	**IL-1 beta**	100	118	1.18
IL1F10	**IL-1 F10**	100	163	1.63
IL1F5	**IL-1 F5**	100	46	-2.17
IL36A	**IL-1 F6**	100	71	-1.41
IL37	**IL-1 F7**	100	41	-2.44
IL36B	**IL-1 F8**	100	119	1.19
IL36G	**IL-1 F9**	100	142	1.42
IL1RAP	**IL-1 R3**	100	121	1.21
IL1RL1	**IL-1 R4**	100	131	1.31
IL1RL2	**IL-1 R6**	100	96	-1.04
IL1RAPL1	**IL-1 R8**	100	192	1.92
IL1RAPL2	**IL-1 R9**	100	119	1.19
IL1RN	**IL-1 ra**	100	63	-1.59
IL1R1	**IL-1 RI**	100	125	1.25
IL1R2	**IL-1 RII**	100	108	1.08
IL10	**IL-10**	100	83	-1.20
IL10RA	**IL-10 R alpha**	100	144	1.44
IL10RB	**IL-10 R beta**	100	191	1.91
IL11	**IL-11**	100	91	-1.10
IL12B	**IL-12 p40**	100	162	1.62
IL12A	**IL-12 p70**	100	116	1.16
IL12RB1	**IL-12 R beta 1**	100	92	-1.09
IL12RB2	**IL-12 R beta 2**	100	114	1.14
IL13	**IL-13**	100	48	-2.08
IL13RA1	**IL-13 R alpha 1**	100	87	-1.15
IL13RA2	**IL-13 R alpha 2**	100	152	1.52
IL15	**IL-15**	100	62	-1.61
IL15RA	**IL-15 R alpha**	100	49	-2.04
IL16	**IL-16**	100	182	1.82
IL17A	**IL-17**	100	99	-1.01
IL17B	**IL-17B**	100	62	-1.61
IL17RB	**IL-17B R**	100	72	-1.39
IL17C	**IL-17C**	100	50	-2.00
IL17D	**IL-17D**	100	49	-2.04
IL25	**IL-17E**	100	103	1.03
IL17F	**IL-17F**	100	49	-2.04
IL17RA	**IL-17R**	100	88	-1.14
IL17RC	**IL-17RC**	100	142	1.42
IL17RD	**IL-17RD**	100	83	-1.20
IL18BP	**IL-18 BPa**	100	119	1.19
IL18R1	**IL-18 R alpha**	100	170	1.70
IL18RAP	**IL-18 R beta**	100	154	1.54
IL19	**IL-19**	100	53	-1.89
IL2	**IL-2**	100	83	-1.20
IL2RA	**IL-2 R alpha**	100	143	1.43
IL2RB	**IL-2 R beta**	100	99	-1.01
IL2RG	**IL-2 R gamma**	100	107	1.07
IL20	**IL-20**	100	105	1.05
IL20RA	**IL-20 R alpha**	100	90	-1.11
IL20RB	**IL-20 R beta**	100	136	1.36
IL21	**IL-21**	100	89	-1.12
IL21R	**IL-21 R**	100	95	-1.05
IL22	**IL-22**	100	66	-1.52
IL22RA2	**IL-22 BP**	100	183	1.83
IL22RA1	**IL-22 R**	100	111	1.11
IL23A	**IL-23**	100	138	1.38
IL23R	**IL-23 R**	100	66	-1.52
IL24	**IL-24**	100	74	-1.35
IL26	**IL-26**	100	80	-1.25
IL28A	**IL-28A**	100	98	-1.02
IFNL3	**IL-28B**	100	109	1.09
IL29	**IL-29**	100	56	-1.79
IL3	**IL-3**	100	138	1.38
IL3RA	**IL-3 R alpha**	100	93	-1.08
IL31	**IL-31**	100	185	1.85
IL33	**IL-33**	100	100	1.00
IL34	**IL-34**	100	119	1.19
IL4	**IL-4**	100	95	-1.05
IL4R	**IL-4 R**	100	54	-1.85
IL5	**IL-5**	100	55	-1.82
IL5RA	**IL-5 R alpha**	100	132	1.32
IL6	**IL-6**	100	56	-1.79
IL6R	**IL-6 R**	100	172	1.72
IL7	**IL-7**	100	61	-1.64
IL7R	**IL-7 R alpha**	100	156	1.56
CXCL8	**IL-8**	100	60	-1.67
IL9	**IL-9**	100	98	-1.02
CCL2	**MCP-1**	100	66	-1.52
CCL8	**MCP-2**	100	93	-1.08
CCL7	**MCP-3**	100	73	-1.37
CCL13	**MCP-4**	100	183	1.83
CCL3	**MIP-1a**	100	55	-1.82
CCL4	**MIP-1b**	100	61	-1.64
CCL15	**MIP-1d**	100	163	1.63
CCL20	**MIP-3 alpha**	100	83	-1.20
TNFA	**TNF-alpha**	100	55	-1.82
TNFB	**TNF-beta**	100	95	-1.05

### HTS Identified Anti-inflammatory and Antibacterial Human Metabolites Against C. difficile

**Figure 1:**
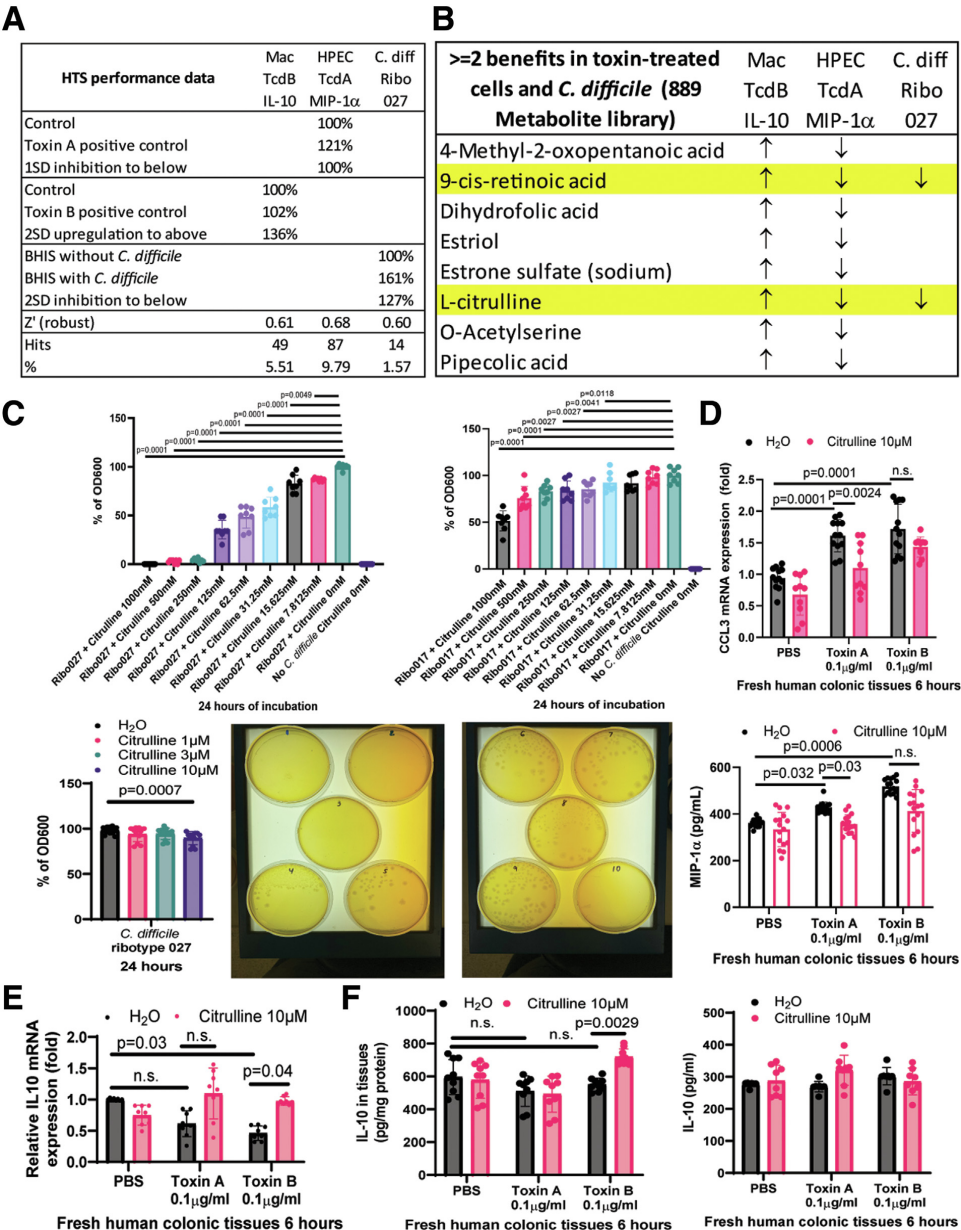
**Citrulline is an anti-inflammatory metabolite.** (*A*) Hit rates and performance data of multidimensional HTS of human metabolites. All assays had robust Z’ factors above 0.5, indicating valid assays. Toxin A induced MIP-1α secretion in HPECs. Toxin B did not affect IL-10 secretion in macrophages. The HTS sought MIP-1α-inhibitory and IL-10-inducing metabolites. *C. difficile* increased absorbance 600 nm reading in BHI broth-filled wells. Antibacterial metabolites reduced the absorbance readings in *C. difficile*-containing wells. (*B*) Human metabolites that increased IL-10 secretion by 2 SD in toxin B-treated macrophages, reduced MIP-1α secretion by 1 SD in toxin A-treated HPECs, and reduced *C. difficile* viability by 2 SD. Data in Table 3, Table 4, Table 5 were compared. Hits with 2 or more benefits are shown in the list. (*C, upper panels*) Determination of MICs of citrulline. *C. difficile* strains, including hypervirulent toxin A+B+ ribotype 027 (ATCC BAA-1805) and hypervirulent toxin A-B+ ribotype 017 (ATCC 43598) at 1 × 10^5^ spores/mL, were incubated with various concentrations of citrulline in BHI broth with 0.1% taurocholate for 24 hours at 37 ^o^C. The viability of *C. difficile* was determined by absorbance at 600 nm. All citrulline treatment groups were compared to the positive control group, which was set as 100%. Citrulline at 250 to 1000 μM eliminated *C. difficile* ribotype 027 in broth. Therefore, the citrulline’s MIC against *C. difficile* ribotype 027 is 250 μM. Citrulline failed to eliminate *C. difficile* ribotype 017 in broth, so its MIC value is unavailable. Results are pooled from 3 experiments (mean ± SD). One-way ANOVAs were used. (*C, lower right panels*) Determination of MBCs of citrulline. The culture broth samples in the MIC determination after 24-hour incubation were added to the agar plates containing BHI with 0.1% taurocholate and further incubated for 48 hours at 37 ^o^C.

Macrophages are dominant immune cells in the intestine that secrete IL-10,^13^^,^^14^ whereas epithelial cells are a prominent component in colonic mucosa that produces MIP-1α.^12^ Gut metabolites drive the disease activity of the *C. difficile*-infected mice,^10^ establishing a premise to utilize HTS to identify human metabolites that affect *C. difficile* levels and cytokine expression in toxin-treated macrophages and colonic epithelial cells. These HTS assays were robust and valid (Figure 1*A*). The hit lists are shown in Table 3, Table 4, Table 5.

**Table:** 

1 Ribotype 027 + citrulline 1000 μM (no colony)	6 Ribotype 017 + citrulline 1000 μM (colonies found)
2 Ribotype 027 + citrulline 500 μM (no colony)	7 Ribotype 017 + citrulline 500 μM (colonies found)
3 Ribotype 027 + citrulline 250 μM (no colony)	8 Ribotype 017 + citrulline 250 μM (colonies found)
4 Ribotype 027 + citrulline 125 μM (colonies found)	9 Ribotype 017 + citrulline 0 μM (colonies found)
5 Ribotype 027 + citrulline 0 μM (colonies found)	10 No *C. difficile* No citrulline (no colony) Citrulline at 250 to 1000 μM eliminated *C. difficile* ribotype 027 on agar plates, so its MBC value is 250 μM. Citrulline 250 to 1000 μM failed to eliminate *C. difficile* ribotype 017 on agar plates, so its MBC value is unavailable. (*C lower left panel*) Antibacterial effect of low concentrations of citrulline. *C. difficile* ribotype 027 at 1 × 10^5^ spores/mL was incubated with various concentrations of citrulline in BHI broth with 0.1% taurocholate for 24 hours at 37 ^o^C. The viability of *C. difficile* was determined by absorbance at 600 nm. All citrulline treatment groups were compared with the positive control group, which was set as 100%. Citrulline at 10 μM mildly inhibited *C. difficile* ribotype 027. Results are pooled from 3 experiments. (*D–F*) Fresh human colonic explants were pretreated with 10 μM citrulline for 30 minutes, followed by incubation with 0.1 mg/mL toxin A or toxin B for 6 or 24 hours. (*D, left*) CCL3 mRNA expression. Real-time RT-PCR showed that citrulline significantly reduced CCL3 mRNA expression in toxin A-treated fresh colonic explants. n = 8–16 patients per group (mean ± SD). One-way ANOVAs were used. (*D, right*) MIP-1α in conditioned media was detected by 27-plex multiplex ELISA. Citrulline inhibited toxin A-mediated MIP-1α secretion. n = 8–16 patients per group (mean ± SD). One-way ANOVAs were used. (*E*) IL10 mRNA expression. Real-time RT-PCR showed that citrulline significantly increased IL10 mRNA expression in toxin B-treated fresh colonic explants. n = 8 patients per group (mean ± SD). One-way ANOVAs were used. (*F*) IL-10 in the tissue lysates and conditioned media was detected by ELISA. Citrulline increased IL-10 levels in toxin B-treated tissues but not in conditioned media. n = 8 patients per group (mean ± SD). One-way ANOVAs were used.

**Table 3:** Drug Screening for Anti-inflammatory Effects in Macrophages

(-)-aspartic acid	Estriol
1,2-Dipalmitoyl-sn-glycerol	Estrone sulfate (sodium)
11-Beta-hydroxyandrostenedioine	Gluconate (calcium)
2',4'-dimethylacetophenone	Glutathione oxidized
2,6-dimethylhydroquinone	Glycocholic acid
2-Hydroxyadipic acid	L-glyceric acid (sodium)
2-methylcyclohexanone	L-gulose
2-oxobutanoic acid	Liothyronine
3-(3-methoxyphenyl) propionic acid	L-citrulline
3-hydroxycapric acid	L-ornithine
3- methylcrotonylglycine	L-thyroxine (sodium salt pentahydrate)
4-Methyl-2-oxopentanoic acid	Meglutol
4- Methylcatechol	Methyl vanillate
5- Methylcytidine	N-acetylputrescine hydrochloride
9-cis-retinoic acid	O-acetylserine
Allantoin	Orotic acid
Asymmetric dimethylarginine	oxytetracycline (hydrochloride)
Cholestenone	Phosphorylcholine
Cholesteryl behenate	Quinolinic acid
Deoxycholic acid sodium salt	R-+-citronellal
Dihydrofolic acid	Retinoic acid
DL-alanine	Sulcatone
DL-tryptophan	Thiamine (hyrdochloride)
D-phenylalanine	Uracil Uric acid

Note: A list of human metabolites that increased IL-10 secretion by 2 standard deviations in toxin B-treated primary human macrophages. The macrophages (5000 cells/well) were pretreated with 10 μM 889 compounds from human endogenous metabolite panels (HY-L030, MedChemExpress), followed by 0.1 mg/mL *C. difficile* toxin B for 6 hours, and the supernatants were collected for IL-10 ELISAs.

ELISA, enzyme-linked immunosorbent assay; IL, interleukin.

**Table 4:** Drug Screening for Anti-inflammatory Effects in HPECs

Human metabolites that decreased MIP-1α expression in toxin A-treated HPECs.	
(-)-(S)-Equol	Estrone sulfate (potassium)
(-)-Limonene	Estrone sulfate (sodium)
(Ethoxymethyl) benzene	FAPy-adenine
1,5-Anhydrosorbitol	Gallic acid (hydrate)
2-(1H-Indol-3-yl) ethan-1-ol	Glycochenodeoxycholic acid
2,3,5-Trimethylpyrazine	Guanosine 5'-diphosphate (disodium salt)
20 (S)-Hydroxycholesterol	Hexadecanedioic acid
2'-Deoxyadenosine monohydrate	Hydroxocobalamin (monohydrochloride)
2'-Deoxyadenosine-5'-triphosphate (trisodium)	L-Alanine
2-Hydroxy-4-methylbenzaldehyde	L-Alloisoleucine
2-Phenylacetamide	L-Cysteinesulfinic acid
3,3'-Diiodo-L-thyronine	L-Dihydroorotic acid
3-Chloro-L-tyrosine	Levoglucosan
3-Indolepropionic acid	Lignoceric acid
3-Methyl-2-cyclopenten-1-one	L-Methionine sulfoxide
3-Methyl-2-oxobutanoic acid	L-citrulline
4- Methyl-2-oxopentanoic acid	L-Ornithine (hydrochloride)
5,6-Dihydrouridine	Methyl 2-(1H-indol-3yl)acetate
5-Hydroxymethyl-2-furancarboxylic acid	N6-Methyladenosine
5- Hydroxytryptophol	N-Acetyl-D-glucosamine
5-Methoxyindole-3-acetic acid	NADP
5-Phenylvaleric acid	NADP (disodium salt)
9-cis-Retinoic acid	Nervonic acid
Acetylcholine (chloride)	Nicotinamide
Acetyl-L-carnitine (hydrochloride)	N-Methylnicotinamide
Aminomalonic acid	Norepinephrine (hydrochloride)
Chenodeoxycholic acid	O-Acetylserine
Choline (chloride)	Octopamine (hydrochloride)
Cinnamoylglycine	p-Anisic acid
Cortisone	Phosphoenolpyruvic acid (tricyclohexylammonium salt)
Cyclic N-Acetyl-D-mannosamine	Phthalic acid mono-2-ethylhexyl ester
Cyclohexaneacetic acid	p-Hydroxycinnamic acid
D(-)-2-Aminobutyric acid	Pipecolic acid
D-Desthiobiotin	Pregnanediol
Dehydroepiandrosterone sulfate (sodium salt)	Prostaglandin E2
D-Erythro-dihydrosphingosine	Pyrroloquinoline quinone
D-Glutamine	S-Adenosyl-L-methionine (disulfate tosylate)
Dihydrofolic acid	Salicyluric acid
D-N-Acetylgalactosamine	Syringic acid
D-Ribose 5-phosphate (disodium)	Tauroursodeoxycholate (dihydrate)
Dulcite	Theophylline
Epsilon-(gamma-glutamyl)-lysine (TFA)	Triacetin
Estriol	Vitamin B12 γ-Cyclodextrin

Note: A list of human metabolites that reduced MIP-1α secretion by 1 standard deviation in toxin A-treated HPECs. The HPECs (5000 cells/well) were pretreated with 10 μM 889 compounds from human endogenous metabolite panels (HY-L030, MedChemExpress), followed by 0.1 mg/mL *C. difficile* toxin A for 6 hours, and the supernatants were collected for ELISAs.

ELISA, enzyme-linked immunosorbent assay; HPEC, human primary colonic epithelial cell; MIP-1α, macrophage inflammatory protein 1 alpha.

**Table 5:** Antibacterial Assays

Metabolites that inhibit *C. difficile* ribotype 027
1-Methyl-L-histidine
5,6 - Dihydrouridine
7-Dehydrocholesterol
9-cis-Retinoic acid
Adenosine-2'-monophosphate
ATP (disodium salt hydrate)
Campesterol
Cholesterol
Cholesteryl oleate
Glycogen, Mussel
L-citrulline
Pipecolic acid
Pyrrole-2-carboxylic acid
Thymine
Uridine 5' - diphosphoglucose (disodium salt)

Note: A list of human metabolites that reduced *C. difficile* ribotype 027 viability by 2 standard deviations. *C. difficile* ribotype 027 at 1 × 10^5^ spores/mL was incubated with 10 μM 889 compounds from human endogenous metabolite panels in brain heart infusion broth with 0.1% taurocholate for 24 hours at 37 ^o^C. The viability of *C. difficile* was determined by absorbance at 600 nm.

### Citrulline is Safe and Effective Against CDI

Two multifunctional metabolites are noteworthy (Figure 1*B*). Citrulline and 9-cis-retinoic acid inhibited *C. difficile*, induced IL-10 secretion in toxin B-treated macrophages, and reduced MIP-1α secretion in toxin A-treated colonic epithelial cells. Interestingly, *C. difficile*-infected mice had reduced fecal levels of citrulline and retinol.^10^

A blood chemistry panel showed no toxicity in mice treated with repeated 4-fold doses of citrulline (Table 6). However, the retinol treatment caused an elevated blood level of aspartate aminotransferase (AST) in mice, suggesting liver toxicity (Table 6). Citrulline, but not retinol, prevented vancomycin-associated CDI recurrence in regular C57BL/6J mice.^10^ Therefore, citrulline is promising for treating CDI.

**Table 6:** Oral Citrulline Treatment Posed No Adverse Effects to Mice

Normal range	ALP 16–200 U/L	AST 6–221 U/L	ALT 22–133 U/L	Albumin 2.6–5.4 g/dL	BUN 2–71 mg/dL	Creatinine 0.1–1.8 mg/dL
Citrulline 40 mg/kg/day for 7 days						
Mean	114	200	46	3.2	18	0.05
SEM	6	6	2	0.1	0.100	0.05
Status	Normal	Normal	Normal	Normal	Normal	Normal
Retinol 40 mg/kg/day for 7 days						
Mean	131	281	87	3.3	19	0.05
SEM	6	62	49	0.2	1	0.05
Status	Normal	High	Normal	Normal	Normal	Normal

Note: Normal C57BL/6J mice were treated with oral citrulline and retinol treatment (40 mg/kg/day for 7 days). The blood was collected for a blood chemistry panel (IDEXX BioAnalytics). The abnormal value is highlighted in yellow. n = 4 mice per group.

ALP, alkaline phosphatase; ALT, alanine aminotransferase; AST, aspartate aminotransferase; BUN, blood urea nitrogen; SEM, standard error of the mean.

High concentrations of citrulline (250–1000 μM) eliminated *C. difficile* ribotype 027, not ribotype 017, with both minimum inhibitory concentration (MIC) and minimum bactericidal concentration (MBC) of citrulline at 250 μM (Figure 1*C*). As the MBC/MIC ratio = 1, citrulline should be bactericidal against *C. difficile* ribotype 027. Even a low concentration of citrulline at 10 μM could mildly inhibit ribotype 027 (Figure 1*C*).

### Citrulline Protected Toxin-exposed Fresh Human Colonic Explants

*C. difficile* toxin A increased CCL3 mRNA expression and MIP-1α secretion in fresh human colonic explants,^11^^,^^12^ which was reversed by citrulline (Figure 1*D*). Interestingly, citrulline augmented tissue IL10 mRNA expression and IL-10 protein levels in the toxin B-exposed fresh human colonic explants (Figure 1*E–F*), even though it was insufficient to affect secreted IL-10 protein levels in the conditioned media (Figure 1*F*). Relatively low concentrations of citrulline were effective in exerting inflammatory effects in cell culture experiments.^15^, ^16^, ^17^ Interestingly, 10 μM citrulline was sufficient to exert both anti-*C. difficile* and anti-inflammatory effects.

**Figure 2:**
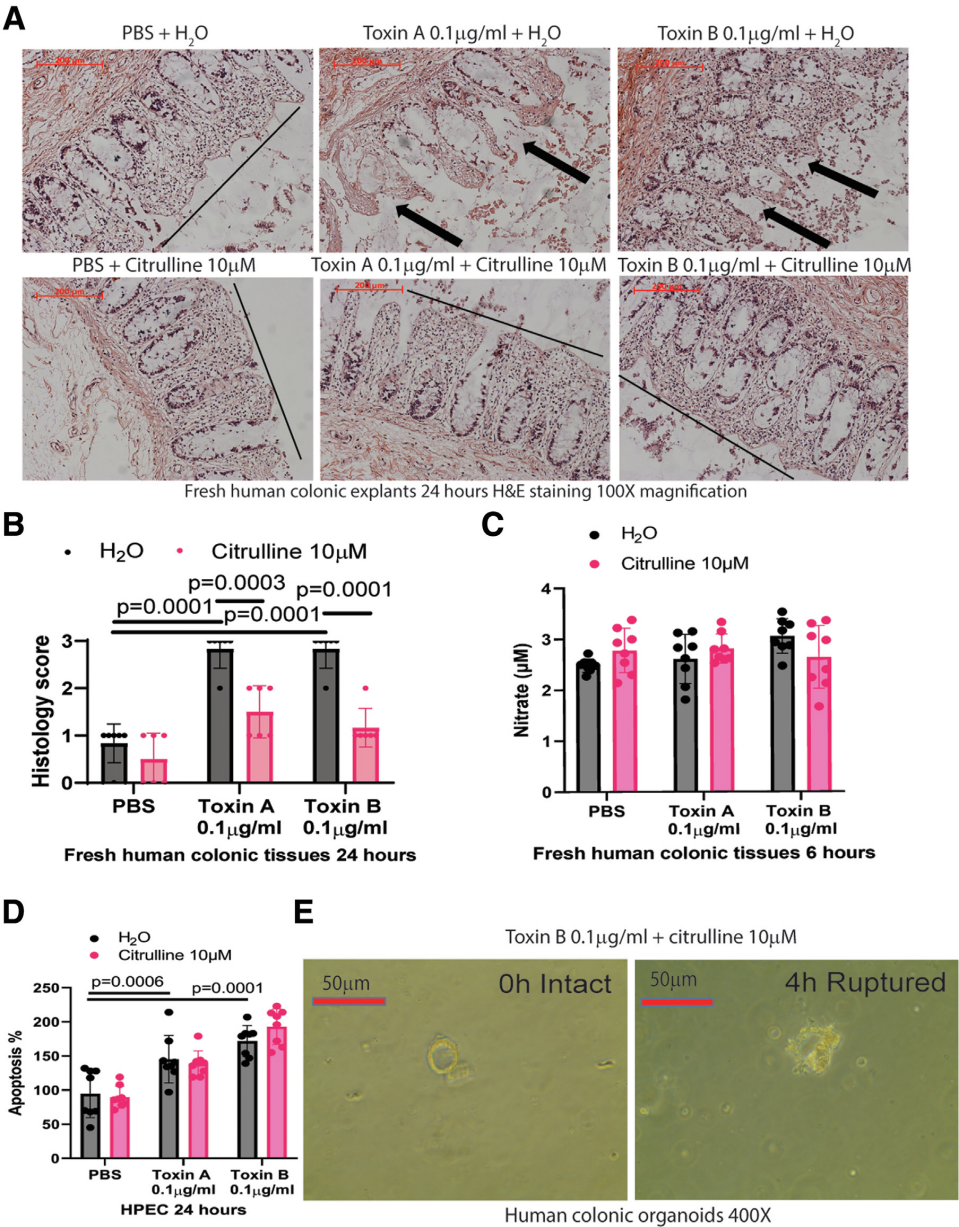
**Citrulline prevented toxin-mediated colonic mucosal injury.** (*A*) Images of H&E-stained fresh human colonic explants. The explants were cultured in serum-free RPMI1640 and treated with or without citrulline and *C. difficile* toxins. Toxins caused mucosal damage, which was prevented by citrulline. 200 mm scale bars are shown in the upper left corners. Results are representative of 6 patients per group. (*B*) Histology scores of colonic explants, which represent mucosal integrity. n = 6 patients per group (mean ± SD). One-way ANOVAs were used. (*C*) After 6-hour incubation with toxins and citrulline, the conditioned media of fresh human colonic explants were collected. Nitrate levels in conditioned media were measured by the nitrate assay. n = 8 patients per group (mean ± SD). One-way ANOVAs were used, but no statistically significant difference was found. (*D*) HPECs were pretreated with citrulline for 30 minutes, followed by incubation with PBS, toxin A, toxin B, and RealTime-Glo Annexin V Apoptosis Assay (JA1000, Promega) reagents for 24 hours.^18^ Results are pooled from 4 experiments (mean ± SD). One-way ANOVAs were used. (*E*) Human colonic organoids from patients with colon cancer were treated with citrulline and toxin B. Six hours later, the organoid in the exact location lost its spherical shape. n = 6 patients.

*C. difficile* toxins disrupt the mucosal lining of fresh human colonic explants.^12^ Citrulline reduced toxin-mediated injury, as reflected by a lowered histology score (Figure 2*A–B*). Citrulline did not affect nitrate levels in the conditioned media of toxin-treated fresh human colonic explants (Figure 2*C*), indicating that citrulline did not affect the nitric oxide (NO) cycle in this condition. Additionally, citrulline did not affect apoptosis in toxin-treated human colonic epithelial cells (Figure 2*D*). Citrulline also failed to prevent toxin B-mediated rupture of human colonic organoids (Figure 2*E*). Thus, citrulline should protect the intestine via NO-independent anti-inflammatory effects but not cytoprotective effects.

### Citrulline Protected Survival Among Hamsters With Primary CDI

**Figure 3:**
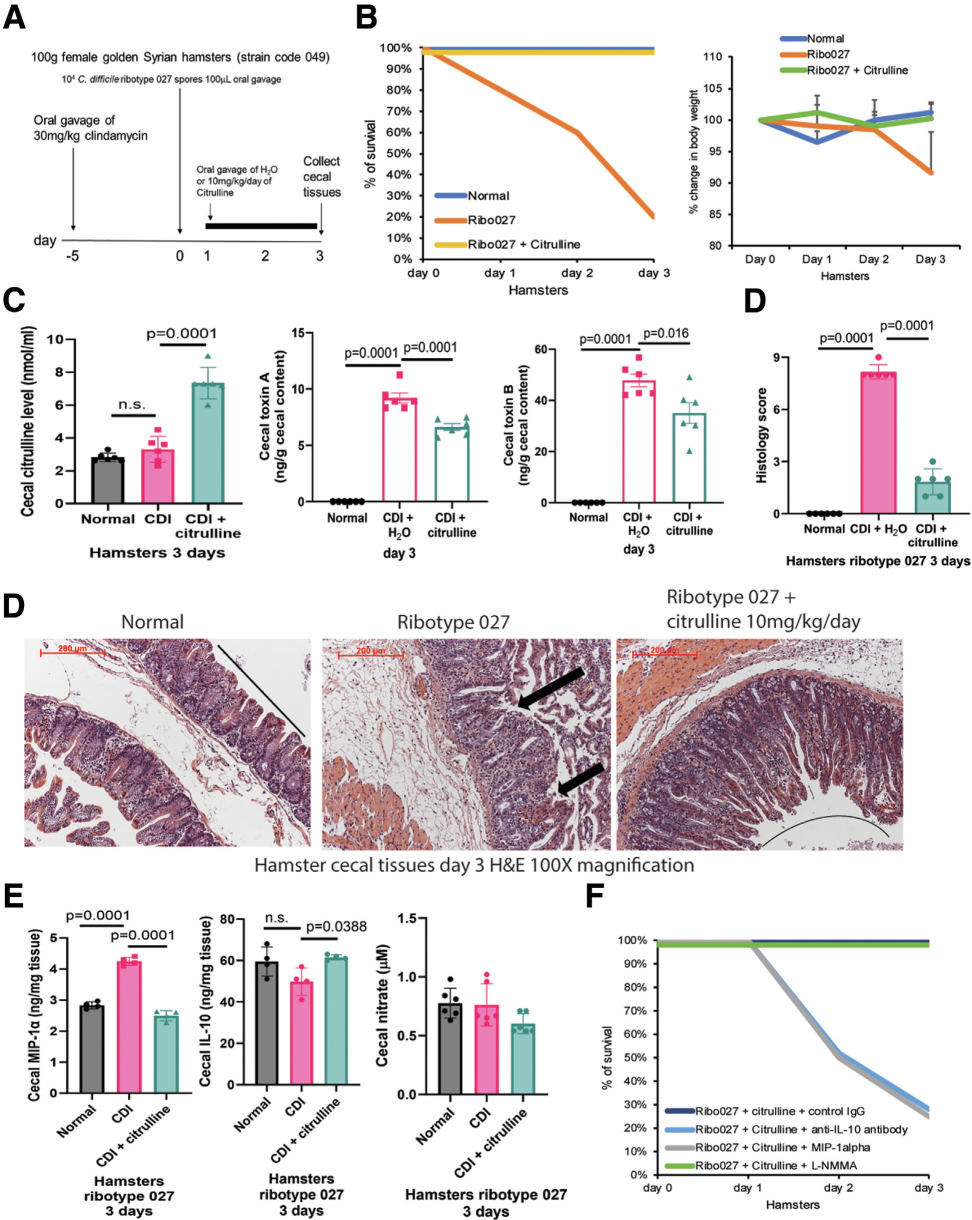
**Oral citrulline treatment protected hamsters against primary CDI with IL-10 and MIP-1α modulation.** (*A*) Experimental plan of primary CDI in regular hamsters. The hamsters were infected with hypervirulent *C. difficile* ribotype 027, followed by oral citrulline treatment on days 1 to 3. (*B*) The survival rate and changes in body weight. 80% of hamsters died on day 3 post-infection, which was prevented by oral citrulline **treatment**. n = 10 hamsters per group. (*C*) Cecal citrulline and toxin levels. Oral citrulline treatment significantly increased cecal citrulline and reduced toxin levels in infected hamsters. n = 6 hamsters per group (mean ± SD). One-way ANOVAs were used. (*D, upper panel*) Histology scores of cecal tissues. n = 6 hamsters per group (mean ± SD). One-way ANOVAs were used. (*D, lower panel*) Images of H&E-stained cecal tissues. Citrulline reduced CDI-associated cecal mucosal injury. 200 mm scale bars are shown in the upper left corners. (*E*) Cecal tissues were taken and homogenized in RIPA buffer with protease inhibitor cocktail. The IL-10 and MIP-1α levels in the cecal homogenates were detected by ELISA. Nitrate levels in the cecal homogenates were measured by the nitrate assays. Citrulline treatment reduced cecal MIP-1α and increased cecal IL-10 but did not affect cecal nitrate levels in infected hamsters. n = 4 hamsters per group (mean ± SD). One-way ANOVAs were used. (*F*) Survival rate. The hamsters were infected with hypervirulent *C. difficile* ribotype 027, followed by oral citrulline treatment. Nitrate synthase inhibitor NG-Monomethyl-L-arginine acetate/L-NMMA (HY-18732A, MedChemExpress) 10 mg/kg/day was fed to the *C. difficile* ribotype 027-infected hamsters via oral gavage from day 1 to day 3. MIP-1α 10 mg/hamster (300-08, PeproTech) and control IgG 100 mg/hamster (AB-108-C, R&D Systems), and anti-IL-10 neutralizing antibodies 100 mg/hamster (AB-217-NA, R&D Systems) were injected intraperitoneally to the infected hamsters once on day 1. n = 8 hamsters per group.

Hypervirulent *C. difficile* ribotype 027 resulted in significant mortality among infected patients in North America.^19^ The survival protection of oral citrulline treatment was assessed using ribotype 027-infected hamsters (Figure 3*A*). CDI cecitis reduced the survival rate to 25% and body weight to 90% on day 3, which was prevented by oral citrulline treatment (Figure 3*B*). Citrulline treatment significantly increased cecal citrulline and reduced cecal *C. difficile* toxin levels in the infected hamsters (Figure 3*C*).

### Oral Citrulline Treatment Mitigated Primary CDI Cecitis in Regular Hamsters With MIP-1α Suppression and IL-10 Induction

Oral citrulline treatment protected regular hamsters against cecitis-associated cecal mucosal disruption, neutrophil infiltration, and bleeding, as reflected by histology scores (Figure 3*D*). Citrulline treatment reduced cecal MIP-1α levels and increased cecal IL-10 levels (Figure 3*E*). A single intraperitoneal injection of MIP-1α and anti-IL-10 neutralizing antibodies abolished the survival protection of citrulline on day 3 post-infection (Figure 3*F*).

### The Protective Effect of Citrulline in Infected Hamsters Was NO-independent

Like the human intestine (Figure 2*C*), citrulline treatment did not affect cecal nitrate levels in the infected hamsters (Figure 3*E*). Oral administration of a nitric oxide synthase (NOS) inhibitor NG-monomethyl-L-arginine acetate (L-NMMA) did not affect the survival protection of citrulline in the infected hamsters (Figure 3*F*). Oral citrulline treatment conferred NO-independent short-term protection against primary CDI in regular hamsters by reducing MIP-1α and increasing IL-10 expression.

### Citrulline and Vancomycin Synergistically Eliminated Cecal C. difficile in Regular Hamsters

**Figure 4:**
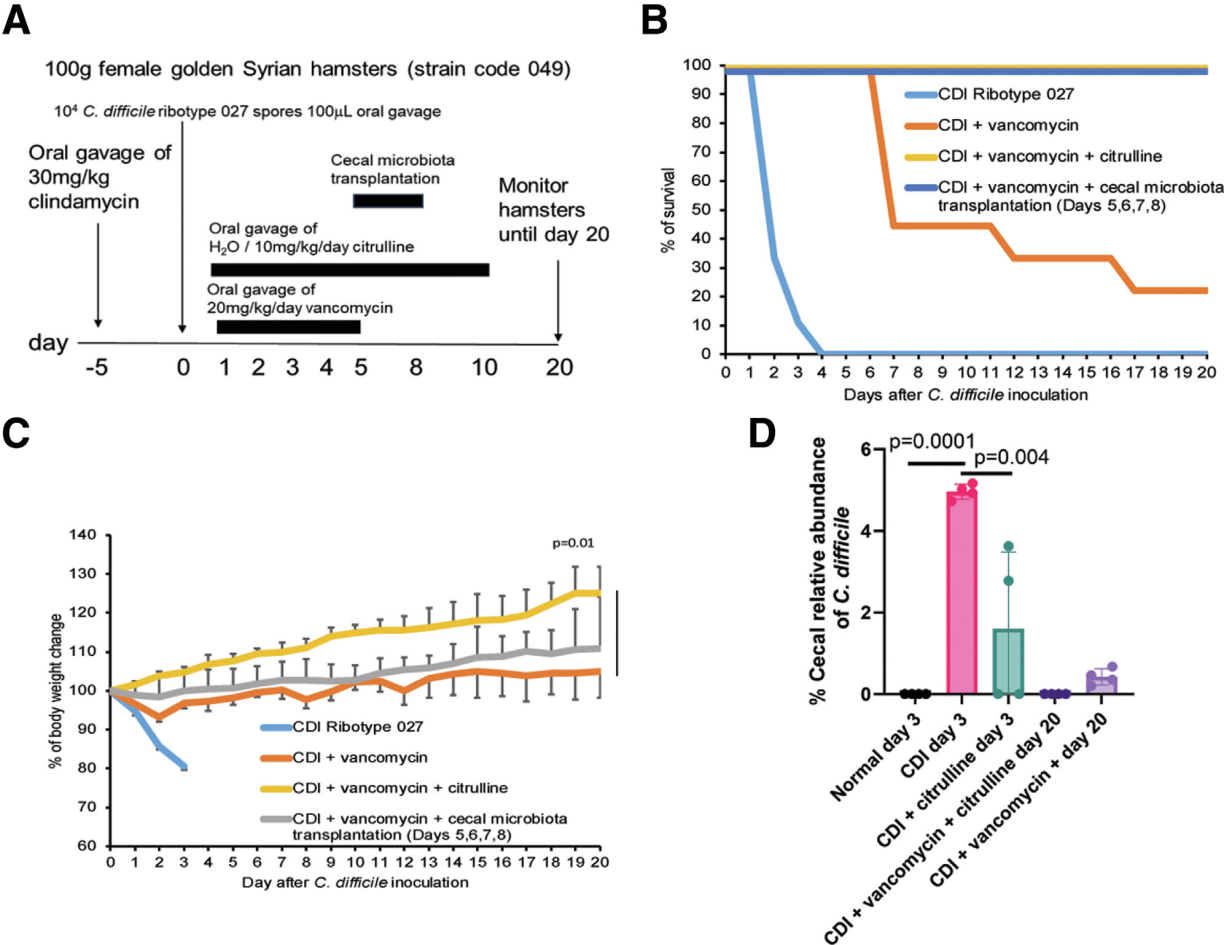
**Oral citrulline and vancomycin treatment synergistically protected hamsters against CDI recurrence.** (*A*) Experimental plan of recurrent CDI in regular hamsters. The *C. difficile* ribotype 027-infected hamsters were orally fed with vancomycin on days 1 to 5. Citrulline was orally fed to the infected hamsters on days 1 to 10. Hamsters were monitored until day 20. (*B*) Survival rate. Vancomycin-treated infected hamsters showed mortality on day 6. Citrulline and CMT prevented CDI recurrence in infected hamsters. Cecal contents from vancomycin- and citrulline-treated donor hamsters were used in the CMT. The recipient hamsters had vancomycin but not citrulline treatment. n = 10 hamsters per group. (*C*) Changes in body weight. n = 10 hamsters per group (mean ± SD). A *t*-test was used. (*D*) Relative cecal *C. difficile* abundance in the hamsters. The microbiome data were generated by CosmosID. Citrulline eliminated *C. difficile* in 2 of 4 hamsters on day 3 and all vancomycin-treated hamsters on day 20. Vancomycin-treated hamsters had low cecal levels of *C. difficile* on day 20. n = 4 hamsters per group (mean ± SD). One-way ANOVA was used.

Consistent with our previous studies,^12^^,^^18^ vancomycin initially protected infected hamsters, but the survival rate was reduced to 20% once treatment was tapered (Figure 4*A–B*). Oral citrulline treatment improved survival and weight gain in the vancomycin-treated infected regular hamsters (Figures 4*B–C*). Intriguingly, transferring the cecal microbiota from vancomycin- and citrulline-treated donor hamsters to vancomycin-treated recipient hamsters (without receiving citrulline treatment) acquired long-term survival protection against CDI recurrence (Figure 4*B–C*).

Shotgun metagenomic sequencing of cecal microbiota revealed that citrulline treatment up to day 3 reduced the abundance of *C. difficile* (Figure 4*D*). Low cecal levels of *C. difficile* were found in vancomycin-treated regular hamsters on day 20, which was further eliminated by citrulline treatment (Figure 4*D*).

**Figure 5:**
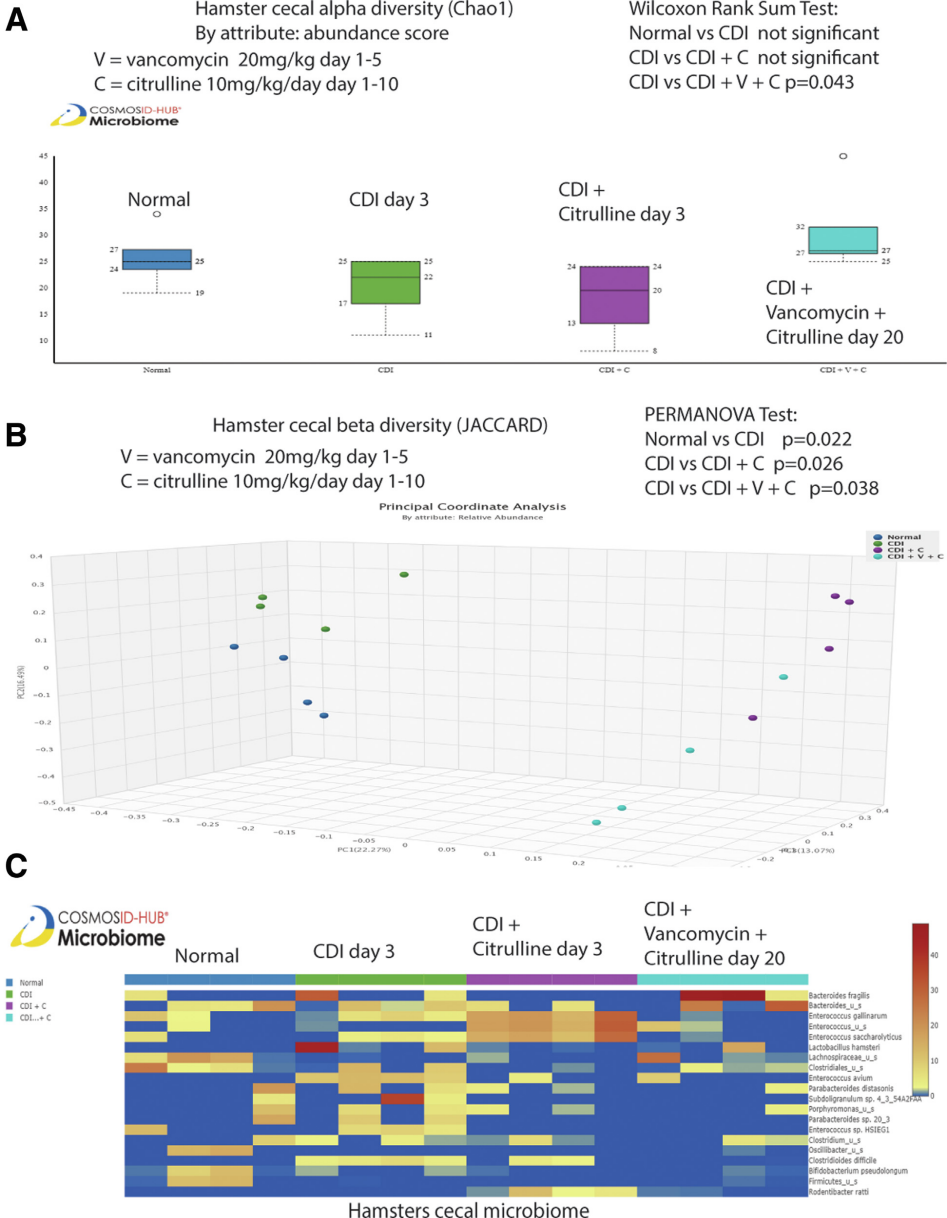
**Citrulline and vancomycin synergistically affected the cecal microbiome in the infected regular hamsters.** (*A–C*) Cecal contents in regular hamsters were collected on days 3 and 20 post-infection. CosmosID performed the sample processing and sequencing. The CosmosID original shotgun 1.0 workflow was used for analysis. (A–B) Alpha and beta diversities of cecal microbiota in regular hamsters are shown. (*C*) Relative abundance of cecal microbiota in regular hamsters is shown. n = 4 hamsters per group. All statistical tests were calculated by CosmosID.

**Figure 6:**
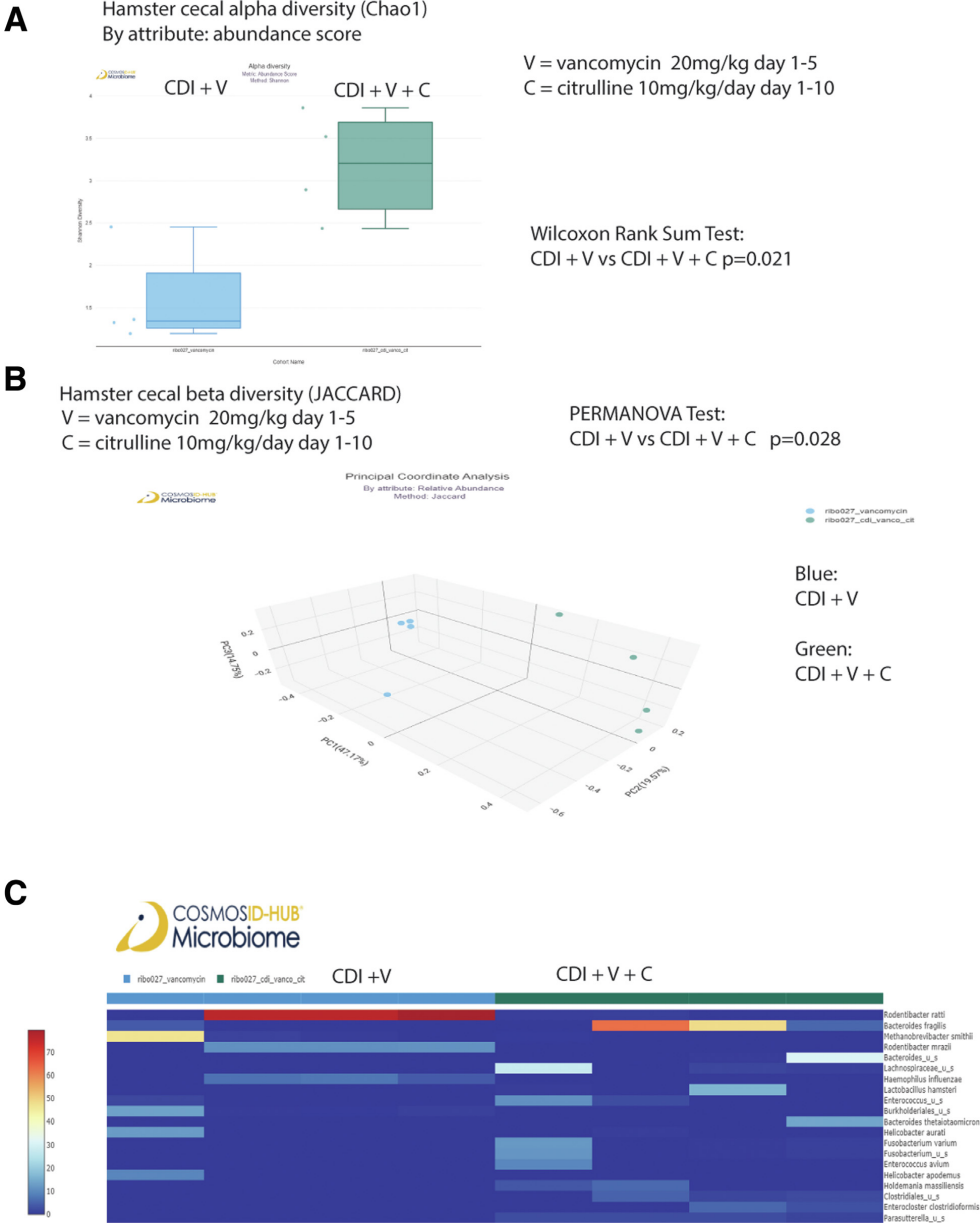
**Citrulline and vancomycin synergistically affected the cecal microbiome in the infected regular hamsters.** (*A–C*) Cecal contents in regular hamsters were collected on day 20 post-infection. CosmosID performed the sample processing and sequencing. A newer CosmosID common kingdom 1.0.0 analysis workflow was used for analysis, which was incompatible with the original shotgun 1.0 workflow. (*A–B*) Alpha and beta diversities of cecal microbiota in regular hamsters are shown. n = 4 hamsters per group. (*C*) Relative abundance of cecal microbiota in regular hamsters is shown. n = 4 hamsters per group. All statistical tests were calculated by CosmosID.

On day 3, short-term citrulline treatment significantly affected beta, but not alpha, diversity (Figure 5*A–B*). On day 20, prolonged 10-day citrulline treatment significantly affected both alpha and beta diversity in vancomycin-treated regular hamsters (Figure 6*A–B*). Citrulline-treated infected hamsters had different relative abundance of bacteria on days 3 and 20 (Figures 5*C* and 6*C*).

### Citrulline Ameliorated Cecitis in Antibiotic-treated Hamsters With Human Fecal Microbiota

**Figure 7:**
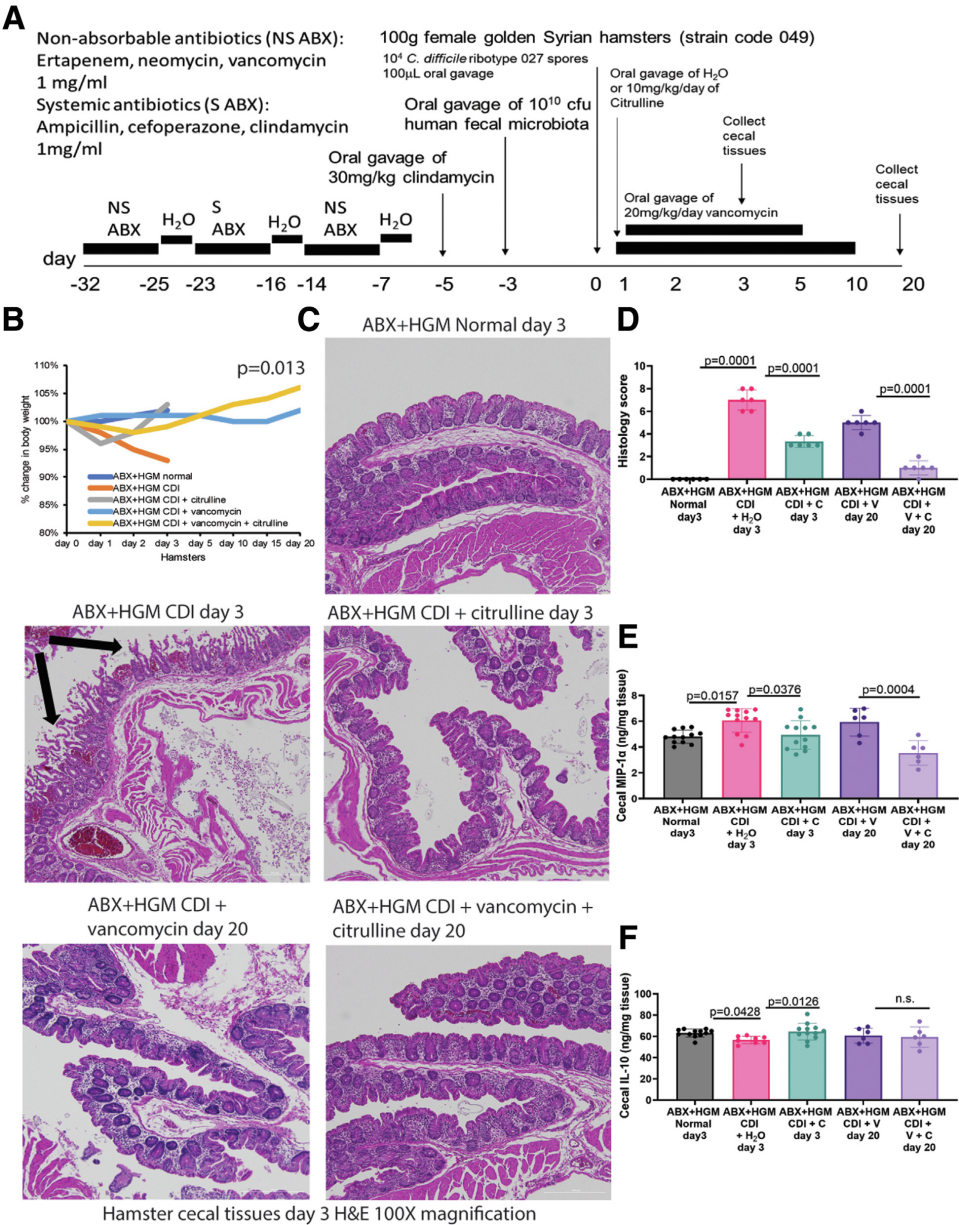
**Oral citrulline treatment protected infected ABX + HGM hamsters.** (*A*) Experimental plan of primary and recurrent CDI in ABX + HGM hamsters. The hamsters were treated with multiple rounds of antibiotics (ABX), followed by inoculation of human gut (fecal) microbiota (HGM) from a healthy donor and *C. difficile* ribotype 027. The infected ABX + HGM hamsters were treated with citrulline from day 1 to days 3 and 10. Some were treated with vancomycin from day 1 to day 10. (*B*) Changes in body weight. The ABX + HGM normal group received antibiotics and human gut microbiota without CDI. All ABX + HGM CDI groups received antibiotics, human gut microbiota, and *C. difficile*. n = 6 ABX + HGM hamsters per group. The difference in body weight change between CDI + vancomycin and CDI + vancomycin + citrulline on day 20 post-infection was statistically significant (*P* = .013). A *t*-test was used. (*C–D*) Images and histology scores of H&E-stained cecal tissues. Citrulline reduced CDI-associated cecal mucosal injury on days 3 and 20. 300 mm scale bars are shown in the lower right corners. n = 6 ABX + HGM hamsters per group (mean ± SD). One-way ANOVA was used. (*E–F*) The cecal levels of MIP-1α and IL-10 were detected by ELISA. Citrulline reduced cecal MIP-1α on days 3 and 20. Citrulline increased cecal IL-10 on day 3 only. C, citrulline; V, vancomycin. n = 6 ABX + HGM hamsters per group (mean ± SD). One-way ANOVAs were used.

The interactions between citrulline and human-like gut microbial environment were evaluated with ABX + HGM hamsters. An established antibiotic regimen combining 6 antibiotics (ABX) was given to deplete endogenous gut microbiota, followed by healthy human donor-derived gut (fecal) microbiota (HGM) engraftment to create a microbiologically humanized hamster model.^20^ The HGM-engrafted hamsters were then inoculated with *C. difficile* ribotype 027 (Figure 7*A*).

All infected ABX + HGM hamsters survived with slight weight loss, substantial cecal injury, increased cecal MIP-1α levels, and slightly decreased cecal IL-10 levels, which were reversed by oral citrulline treatment (Figure 7*B–F*).

After tapering vancomycin, the infected ABX + HGM hamsters survived but presented with low weight gain and moderate cecal injury by day 20 (Figure 7*B–D*). Oral citrulline treatment prevented CDI recurrence with significantly increased weight gain, reduced cecal injury, and lowered cecal MIP-1α levels without affecting cecal IL-10 levels in vancomycin-treated ABX + HGM hamsters through day 20 (Figure 7*B–F*).

**Figure 8:**
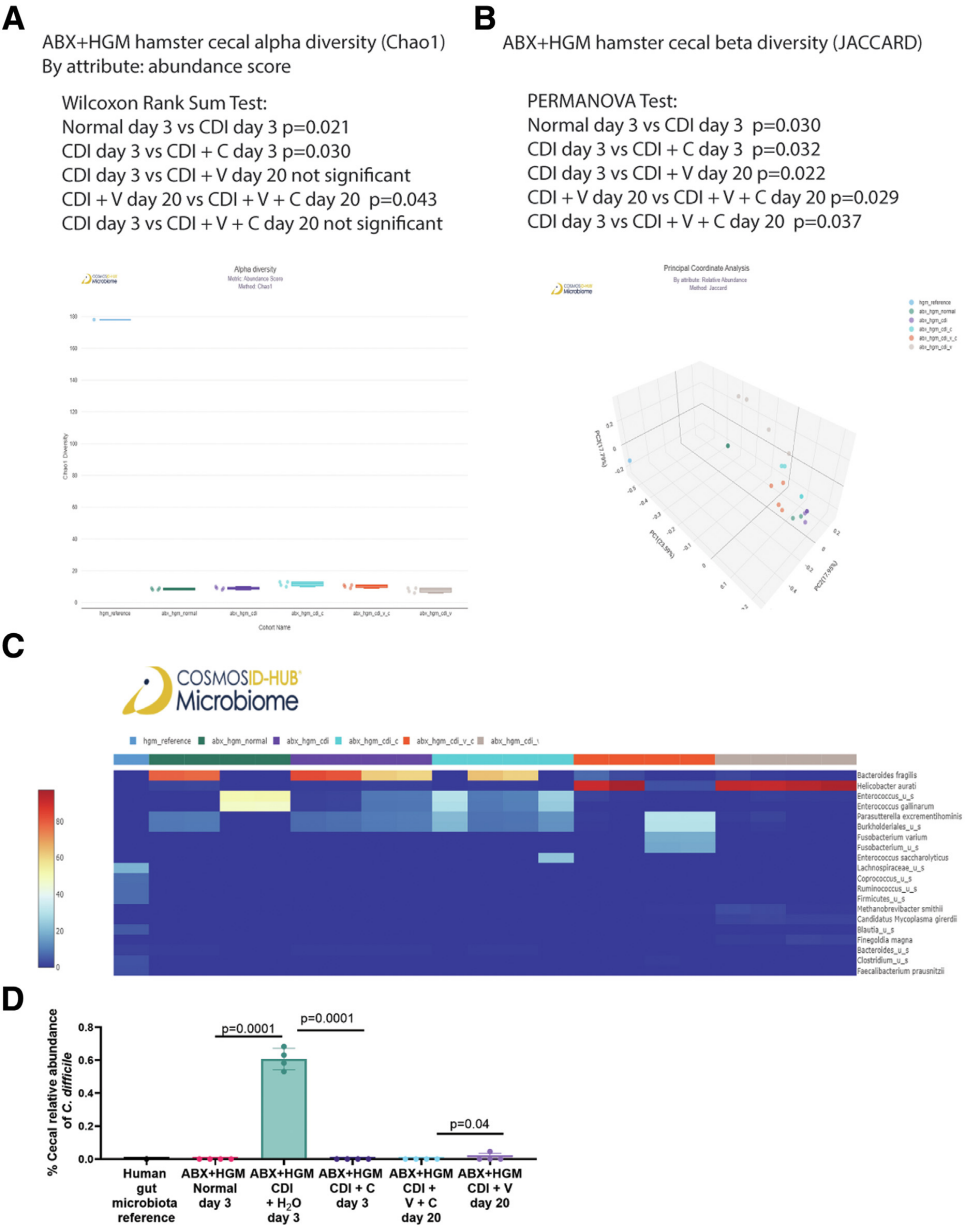
**Citrulline eliminated cecal *C. difficile* in ABX+HGM hamsters.** (*A–D*) Cecal contents in ABX + HGM hamsters were collected on days 3 and 20 post-infection. The fecal microbiota of a human donor was included for reference. CosmosID performed the sample processing and sequencing. A newer CosmosID common kingdom 1.0.0 analysis workflow was used for analysis. (*A–B*) Alpha and beta diversities of cecal microbiota. n = 4 ABX + HGM hamsters per group. (*C*) A heatmap of the relative abundance of cecal bacteria. The relative abundance of *C. difficile* was too low to be shown in the top 20 bacteria list. n = 4 ABX + HGM hamsters per group. (*D*) Relative cecal *C. difficile* abundance. Citrulline treatment eliminated cecal *C. difficile* in infected hamsters on day 3 and vancomycin-treated infected hamsters on day 20. A low cecal level of *C. difficile* was found in vancomycin-treated infected hamsters on day 20. C, citrulline; V, vancomycin. Mean ± SD. n = 4 ABX + HGM hamsters per group. All statistical tests were calculated by CosmosID.

With additional antibiotic treatment, ABX + HGM hamsters had relatively lower cecal alpha diversity than regular hamsters with or without CDI and citrulline treatment (Figures 5*A* and 8*A*). Citrulline treatment slightly increased cecal alpha diversity and affected the relative abundance of bacteria on days 3 and 20 (Figure 8*A* and 8*C*). Citrulline treatment also altered cecal beta diversity in vancomycin-treated infected ABX + HGM hamsters on day 20 (Figure 8*B*).

ABX + HGM hamsters had a much lower cecal abundance of *C. difficile* than regular hamsters (Figures 4*D* and 8*D*), which might be associated with mild colitis and the lack of mortality of infected ABX + HGM hamsters. Citrulline treatment eliminated cecal *C. difficile* in the ABX + HGM hamsters on day 3 and in the vancomycin-treated ABX + HGM hamsters on day 20 (Figure 8*D*).

### Citrulline Immunomodulated Toxin B-mediated Colitis in the Infected Humanized HuCD34-NCG Mice

**Figure 9:**
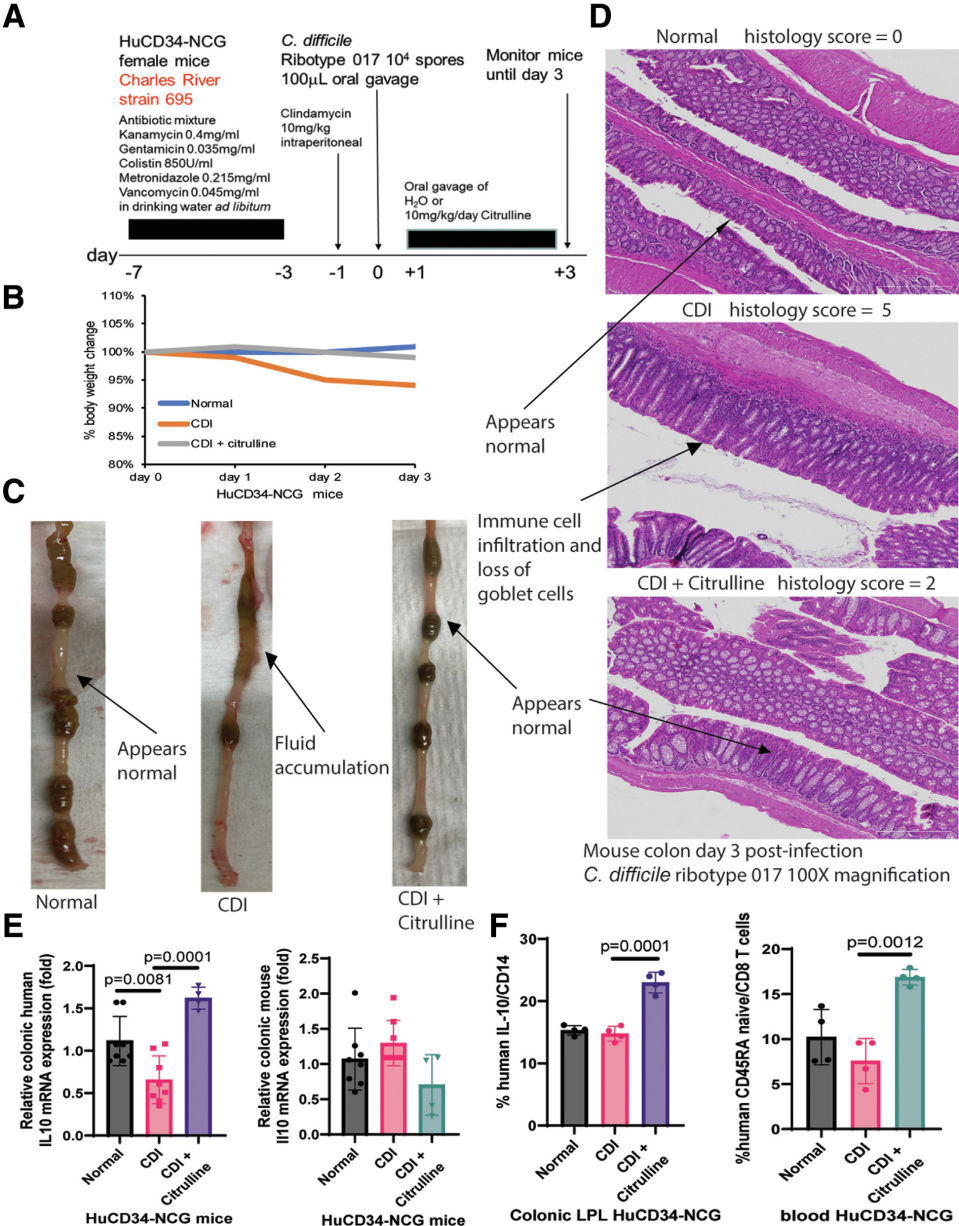
**Citrulline protected against toxin B-mediated colitis in infected immunologically humanized mice.** (*A*) Experimental plan of primary CDI in HuCD34-NCG mice. The HuCD34-NCG mice were infected with toxin B-expressing *C. difficile* ribotype 017 on day 0, followed by oral citrulline treatment on days 1 to 3. The mice were monitored until day 3 post-infection. (*B*) Changes in body weight. The infected HuCD34-NCG mice had mild weight loss, which was prevented by citrulline treatment. n = 4 HuCD34-NCG mice/group. *t*-tests were used, but no statistically significant difference was found. (*C*) Gross morphology of colons on day 3 post-infection. n = 4 HuCD34-NCG mice/group. (*D*) Images of H&E-stained colonic tissues. 300 mm scale bars are shown in the lower right corners. n = 4 HuCD34-NCG mice/group. (*E*) Colonic tissues were collected on day 3 post-infection. Total RNA was extracted and processed for real-time RT-PCR, as described in the Materials and Methods section. Colonic human IL10 and mouse Il10 mRNA expression. Human IL10 was normalized to 18S rRNA. Mouse Il10 was normalized to Gapdh. Citrulline increased colonic human IL10 but not mouse il10 mRNA expression. n = 4 HuCD34-NCG mice/group (mean ± SD). One-way ANOVAs were used. (*F*) Human cells in the colonic LPL and blood on day 3 post-infection were determined using an Attune NxT flow cytometer (ThermoFisher). The cell staining protocol was modified from our previous report.^11^ Human IL-10 expression in CD14 cells was determined with human-specific anti-IL10 (46-7108-41) and anti-CD14 (25-0149-41) antibodies from ThermoFisher. Human naïve CD8 T cells were identified with human-specific anti-CD45RA (25-0458-42) and human anti-CD8A (H003T03B09) antibodies from ThermoFisher. A live/dead fixable green dead cell stain kit (L34969, ThermoFisher) was used to identify live cells. n = 4 HuCD34-NCG mice/group (mean ± SD). One-way ANOVAs were used.

Toxin B is the most clinically crucial pathogenic factor in CDI.^6^ Our laboratory established a new platform to study toxin B pathology using immunologically humanized HuCD34-NCG mice (Figure 9*A*).^11^ The toxin B-expressing *C. difficile* ribotype 017-infected humanized mice exhibited mild weight loss without mortality (Figure 9*B*). They displayed colitis with fluid accumulation, immune cell accumulation, and some loss of goblet cells in the colon, as reflected by increased histology scores (Figure 9*C–D*).

Oral citrulline treatment improved CDI colitis (Figure 9*C–D*) and restored the colonic human IL10 mRNA expression (Figure 9*E*, *left panel*). As the mouse immune system in HuCD34-NCG mice was suppressed, there were no changes in colonic mouse Il10 mRNA expression (Figure 9*E*, *right panel*). Citrulline modulated human immune cell responses to toxin B by augmenting human IL-10 expression within human CD14+ cells (supposedly macrophages) in the colonic lamina propria lymphocyte (LPL) compartment and increasing circulating naïve CD8 cytotoxic T cells in the infected humanized mice (Figure 9*F*).

### Oral Citrulline Treatment Protected Regular Mice Against CDI With Toxin A-dependent MIP-1a Suppression

**Figure 10:**
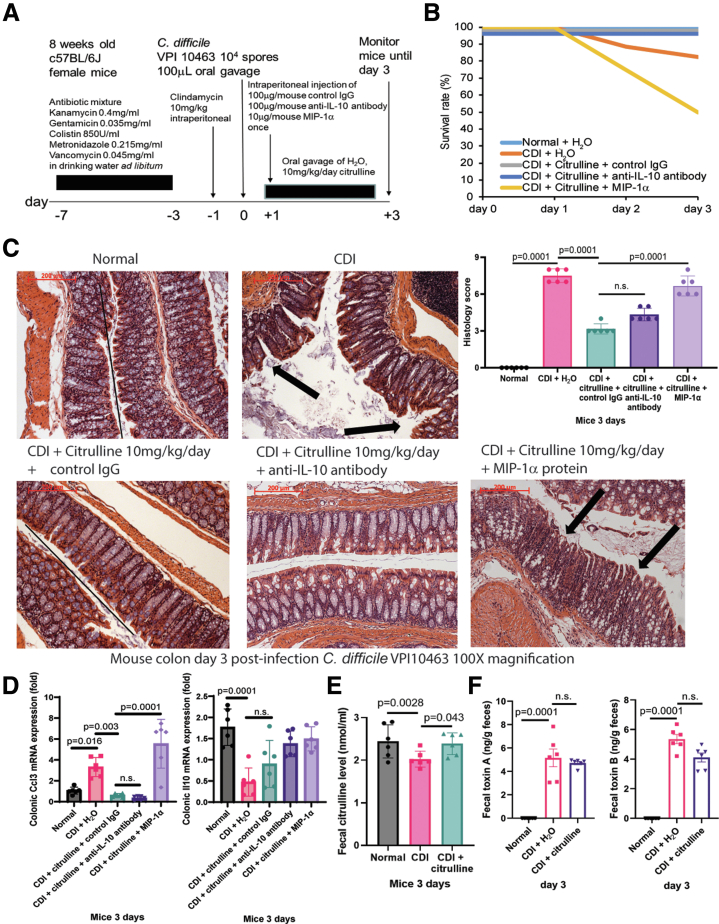
**Citrulline protected mice against primary CDI with MIP-1α inhibition.** (*A*) Experimental plan of primary CDI in regular mice. Mice were infected with *C. difficile* VPI10463. Some mice were intraperitoneally injected with 100mg/mouse control IgG (AB-108-C, R&D Systems), 10 mg/mouse MIP-1α (250-09, PeproTech), and 100 mg/mouse anti-IL-10 neutralizing antibody (AB-417-NA, R&D Systems) once. n = 12 regular mice per group. (*B*) Survival rate. CDI reduced survival rate, which was prevented by citrulline treatment. n = 6 regular mice per group. (*C, left panel*) Images of H&E-stained colonic tissues in infected mice. 200 mm scale bars are shown in the upper left corners. (*C, right panel*) Histology scores. Citrulline reduced CDI-dependent colonic injury, which was reversed by MIP-1α but not anti-IL-10 neutralizing antibody injection. n = 6 regular mice per group (mean ± SD). One-way ANOVAs were used. (*D*) Colonic tissues were collected on day 3 post-infection. Total RNA was extracted and processed for real-time RT-PCR, as described in the Materials and Methods section. Colonic Ccl3 and Il10 mRNA expression were determined by real-time RT-PCR. n = 6 regular mice per group (mean ± SD). One-way ANOVAs were used. (*E–F*) Fecal samples were collected on day 3 post-infection and suspended in ice-cold PBS. After centrifugation at 2000 g for 5 minutes to remove debris, the clear supernatants were used for citrulline assay and *C. difficile* toxin ELISAs. Fecal citrulline and *C. difficile* toxin levels. Oral citrulline treatment increased fecal citrulline but not toxin levels in the infected regular mice. n = 6 regular mice per group (mean ± SD). One-way ANOVAs were used.

In regular mice, toxin A is much more potent than toxin B in evoking cecal and colonic injury and colonic MIP-1α (not IL-10) secretion.^12^^,^^21^ As citrulline prevented mortality and weight loss among mice with CDI colitis,^10^ infected regular mice were used to determine the involvement of MIP-1α in the protective mechanism of citrulline (Figure 10). Primary CDI colitis caused mortality and colonic injury, characterized by mucosal disruption and neutrophil infiltration, as reflected by increased histology score and colonic Ccl3 mRNA expression (Figure 10*B–D*).^11^^,^^12^^,^^18^ Oral citrulline treatment prevented mortality and reduced colonic injury, histology score, and Ccl3 mRNA expression without affecting Il10 mRNA expression (Figure 10*C–D*). A single intraperitoneal injection of MIP-1α, but not anti-IL-10 neutralizing antibodies, abolished the protective effect of citrulline in the infected mice (Figure 10*B–C*).

**Figure 11:**
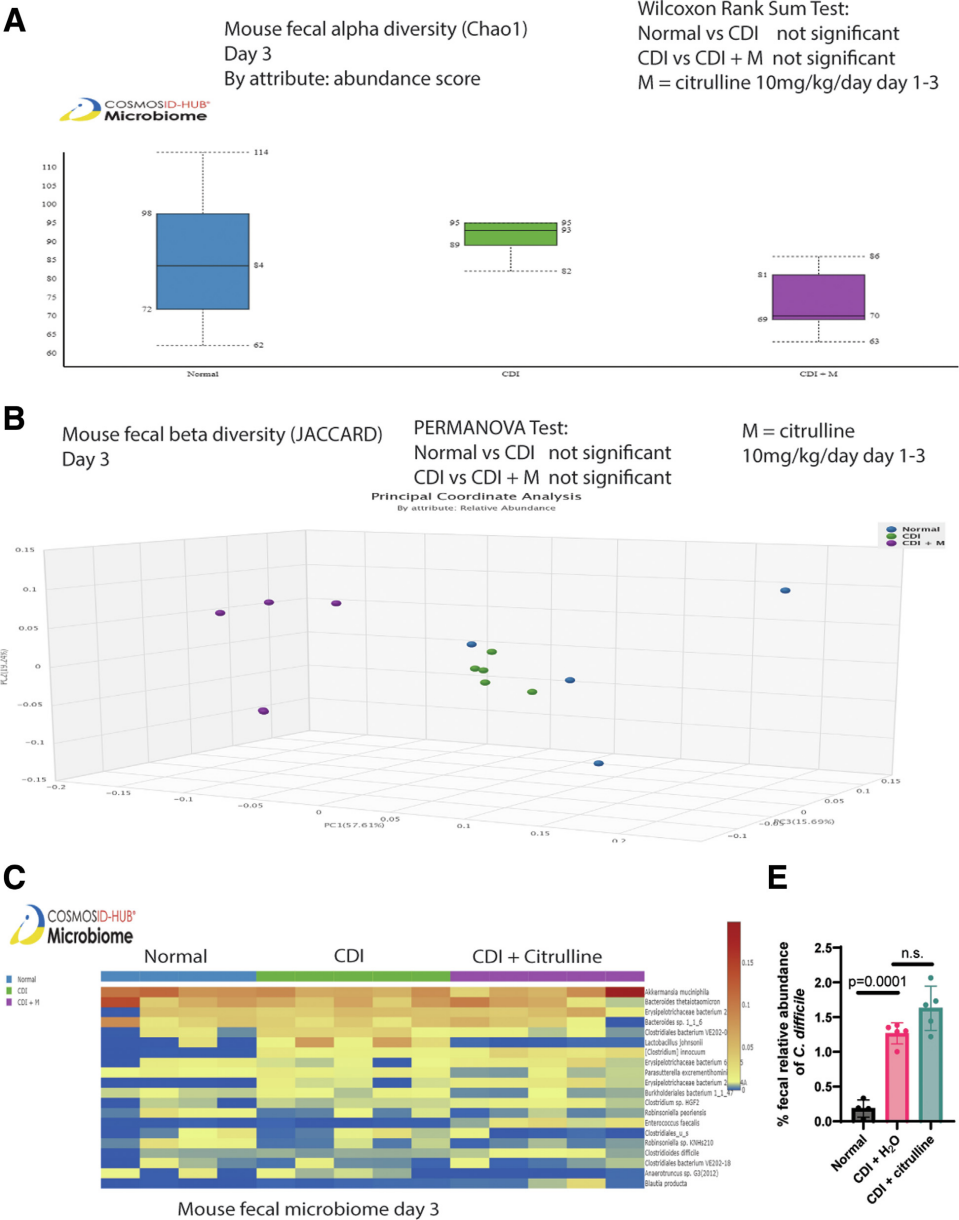
**Citrulline did not affect the fecal microbiome in the infected regular mice.** (*A–C*) Feces from regular mice were collected on day 3 post-infection. CosmosID performed the sample processing and sequencing. The CosmosID original shotgun 1.0 workflow was used for analysis. (*A–B*) Alpha and beta diversities of fecal microbiota. (*C*) A heatmap of the relative fecal abundance of bacteria. The top 20 bacteria are shown. (*D*) Relative fecal *C. difficile* abundance. Oral citrulline treatment did not affect fecal *C. difficile* abundance. Mean ± SD. This microbiome study included 4 normal mice, 5 CDI mice, and 5 CDI + citrulline mice. All statistical tests were calculated by CosmosID.

Citrulline treatment increased fecal citrulline levels but did not affect fecal toxin levels, alpha and beta diversity, and *C. difficile* abundance in the infected mice (Figures 10*E–F* and 11*A–D*). Like HuCD34-NCG mice, citrulline treatment increased circulating naïve CD8 cytotoxic T cells in the infected regular mice (Table 7).

**Table 7:** Oral Citrulline Treatment Enhanced CD8 naïve T Cells in Infected Mice

Flow cytometry of whole blood	(%)	Lymphocytes of total cells	CD8+ Naive T cells	CD8+ Memory T cells	CD8+ of TCRγΔ- CD3+ T cells
Normal	Mean	78.5	54.3	44.8	38.8
	SEM	6.7	3.1	2.9	2.5
CDI	Mean	90.4	47.8	50.8	37.6
	SEM	3.1	1.2	1.4	0.7
CDI + citrulline	Mean	88.0	57.0	41.7	42.0
	SEM	2.3	2.3	2.2	0.7
CDI vs CDI + citrulline		n.s.	*P* = .0121	*P* = .0127	*P* = .0038

Note: Flow cytometry data. C57BL/6J mice were infected with *C. difficile* VPI10463 on day 0. On day 3 post-infection, blood samples were collected and phenotyped by UCLA Immune Assessment Core.^10^ Only statistically significant findings are shown. n = 4 mice per group. One-way ANOVAs were used.

ANOVA, analysis of variance; CDI, *Clostridioides difficile* infection; SEM, standard error of the mean; TCRγΔ, T-cell receptor gamma delta.

### Citrulline Increased HSP27 Phosphorylation and IL-10 Secretion in Toxin B-treated Macrophages

**Figure 12:**
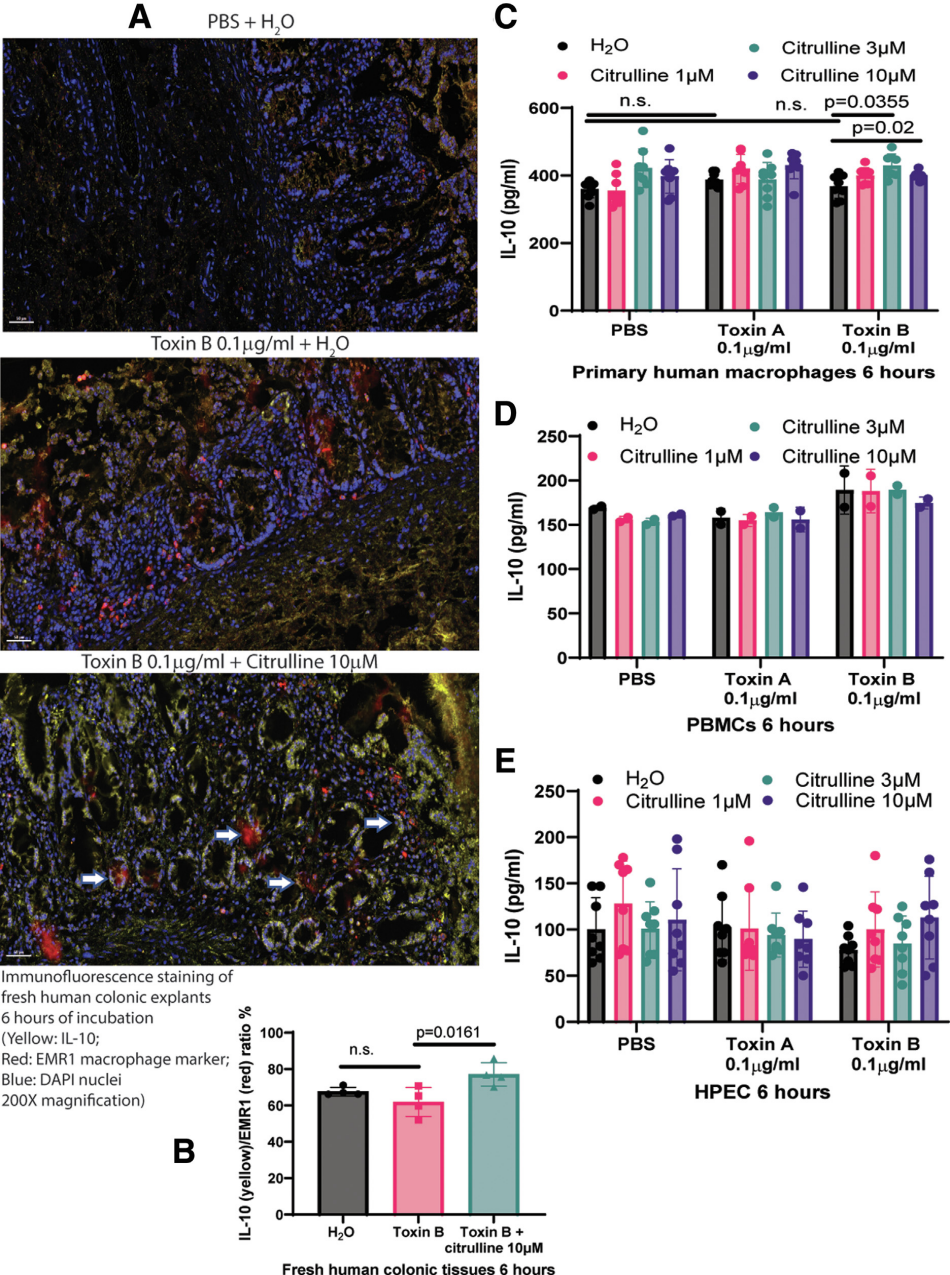
**Citrulline promoted IL-10 expression in toxin B-treated macrophages.** (*A*) Immunofluorescence staining. The fresh human colonic explants were pretreated with 10 μM citrulline for 30 minutes, followed by incubation with 0.1 mg/mL toxin B for 6 hours. Detailed immunofluorescence staining protocol was described in the Materials and Methods section. The control and toxin B-treated groups had relatively low IL-10 expression near macrophages. As indicated by *arrows*, IL-10 expression was increased near macrophages in the toxin B- and citrulline-treated fresh human colonic tissues. The 50 mm scale bar is located at the lower-left corner of each image. n = 4 patients per group (mean ± SD). One-way ANOVA was used. (*B*) Relative IL-10 protein expression in the fresh human colonic expression. Based on the immunofluorescence staining signals, citrulline increased IL-10 fluorescent signal intensity in the EMR1+ macrophages of toxin B-treated fresh human colonic explants. n = 4 patients per group (mean ± SD). One-way ANOVA was used. (*C*) IL-10 levels in conditioned media. Primary human macrophages were pretreated with citrulline for 30 minutes and then incubated with toxins for 6 hours. IL-10 levels were measured by ELISA. Results were pooled from 4 experiments (mean ± SD). One-way ANOVA was used. (*D*) IL-10 levels in conditioned media. PBMCs were pretreated with citrulline for 30 minutes and then incubated with toxins for 6 hours. IL-10 levels were measured by ELISA. Results were pooled from 2 experiments from 2 donors (mean ± SD). One-way ANOVA was used. (*E*) IL-10 levels in conditioned media. HPECs were pretreated with citrulline for 30 minutes and then incubated with toxins for 6 hours. IL-10 levels were measured by ELISA. Results were pooled from 4 experiments (mean ± SD). One-way ANOVA was used.

The source of citrulline- and toxin B-dependent cytokines in the human intestine was identified by immunofluorescence staining. Citrulline-mediated IL-10 protein was located near EMR1-expressing macrophages in toxin B-treated fresh human colonic explants (Figure 12*A–B*). Similarly, citrulline (3–10 μM) induced IL-10 secretion in toxin B-treated primary human macrophages but not primary human peripheral blood mononuclear cells (PBMCs) and human primary colonic epithelial cells (HPECs) (Figure 12*C–E*).

**Figure 13:**
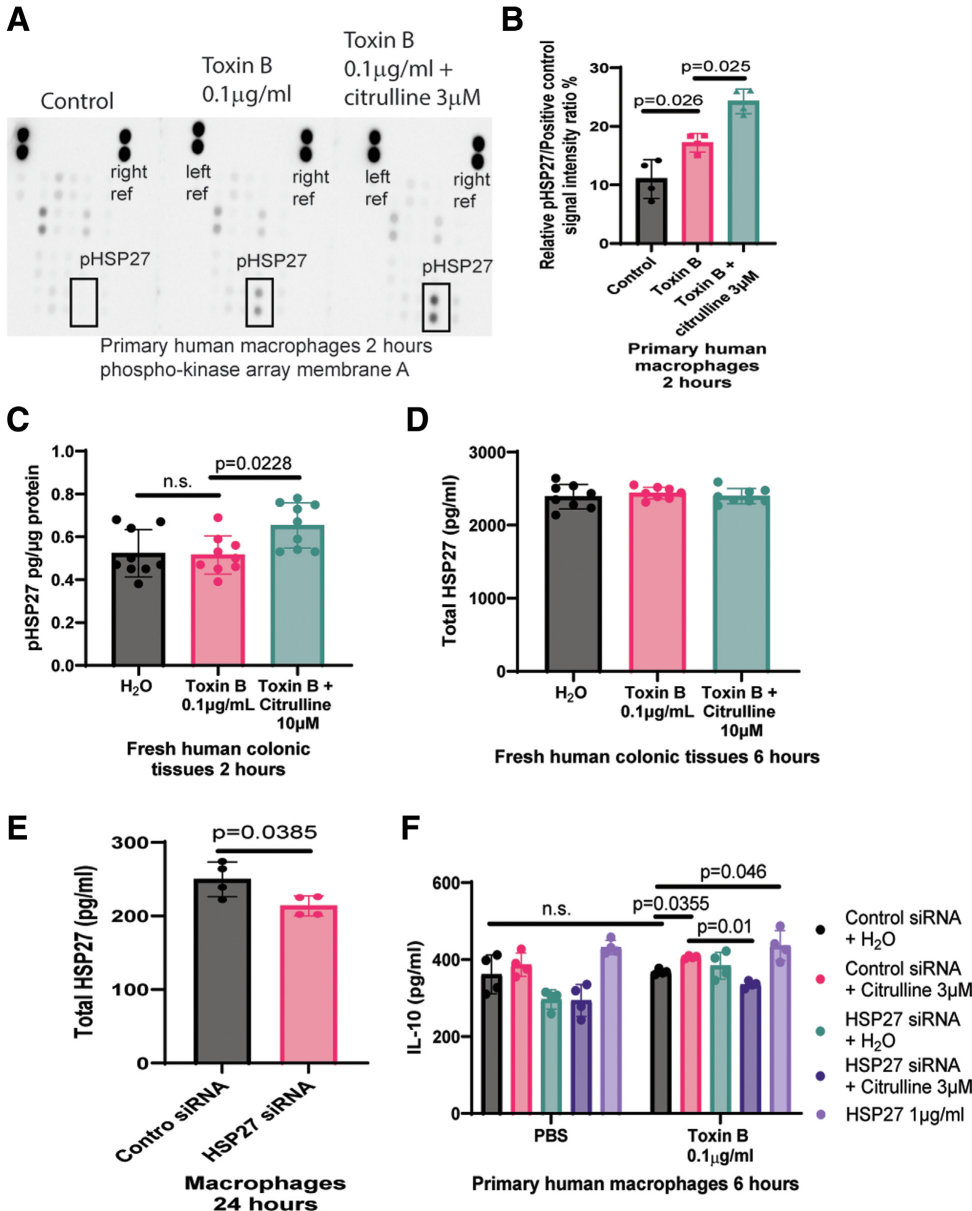
**Citrulline promoted IL-10 expression with HSP27 phosphorylation in toxin B-treated macrophages.** (*A–B*) Phospho-kinase array. Primary human macrophages were treated with or without citrulline for 30 minutes, followed by either PBS or toxin B for 2 hours. The cells were lysed, and the lysates were assayed with the protein Proteome Profiler Human Phospho-Kinase Array Kit (ARY003C, R&D Systems). The images were captured by the Bio-Rad ChemiDoc Imaging system and analyzed by Bio-Rad Image Lab software. The cell lysates were collected for the protein arrays. Citrulline increased phosphorylated HSP27 levels in toxin B-treated macrophages. Results were pooled from 4 experiments (mean ± SD). One-way ANOVA was used. (*C*) Phosphorylated HSP27 levels. The fresh human colonic explants were pretreated with 10 μM citrulline for 30 minutes, followed by incubation with 0.1 mg/mL toxin B for 2 hours. The phosphorylated HSP27 levels were determined by ELISA. Results were pooled from 4 tissue donors (mean ± SD). One-way ANOVA was used. (*D*) Total HSP27 levels. The fresh human colonic explants were pretreated with 10 μM citrulline for 30 minutes, followed by 6 hours of incubation with 0.1 μg/mL toxin A or toxin B. Total HSP27 levels in conditioned media were measured by ELISA. Results were pooled from 4 tissue donors (mean ± SD). One-way ANOVA was used, but no statistically significant difference was found. (*E–F*) Macrophages were transiently transfected with control siRNA (sc-37007) or HSP27 siRNA (sc-29350) from Santa Cruz Biotechnology overnight. (*E*) Total HSP27 ELISA. After overnight transfection, the macrophages were incubated with serum-free RPMI1640 media for 24 hours. Secreted total HSP27 levels of transfected macrophages were measured by ELISA. Results were pooled from 4 experiments (mean ± SD). One-way ANOVA was used. (*F*) IL-10 ELISA. Macrophages (with or without siRNA transfection) were pretreated with citrulline or recombinant human HSP27 (1580-HS-050, R&D Systems) for 30 minutes and incubated with toxin B for 6 hours. IL-10 levels in conditioned media were detected by ELISA. Results were pooled from four experiments (mean ± SD). One-way ANOVA was used.

Phospho-kinase protein array and enzyme-linked immunosorbent assay (ELISA) revealed that citrulline increased phosphorylated HSP27 levels in toxin B-treated macrophages and fresh human colonic explants (Figure 13*A–C*). However, toxin B and citrulline did not affect HSP27 secretion in the fresh human colonic explants (Figure 13*D*).

Transfection of HSP27 small interfering RNA (siRNA) significantly reduced HSP27 secretion in macrophages (Figure 13*E*). siRNA knockdown of HSP27 abolished citrulline-induced IL-10 secretion in toxin B-treated macrophages (Figure 13*F*). Exogenous HSP27 enhanced IL-10 secretion in toxin B-treated macrophages (Figure 13*F*). Therefore, citrulline-dependent intracellular HSP27 phosphorylation might mediate IL-10 secretion in toxin B-treated macrophages.

### Citrulline Caused GSK3a/b Dephosphorylation and Prevented MIP-1α Secretion in Toxin A-treated Colonic Epithelial Cells

**Figure 14:**
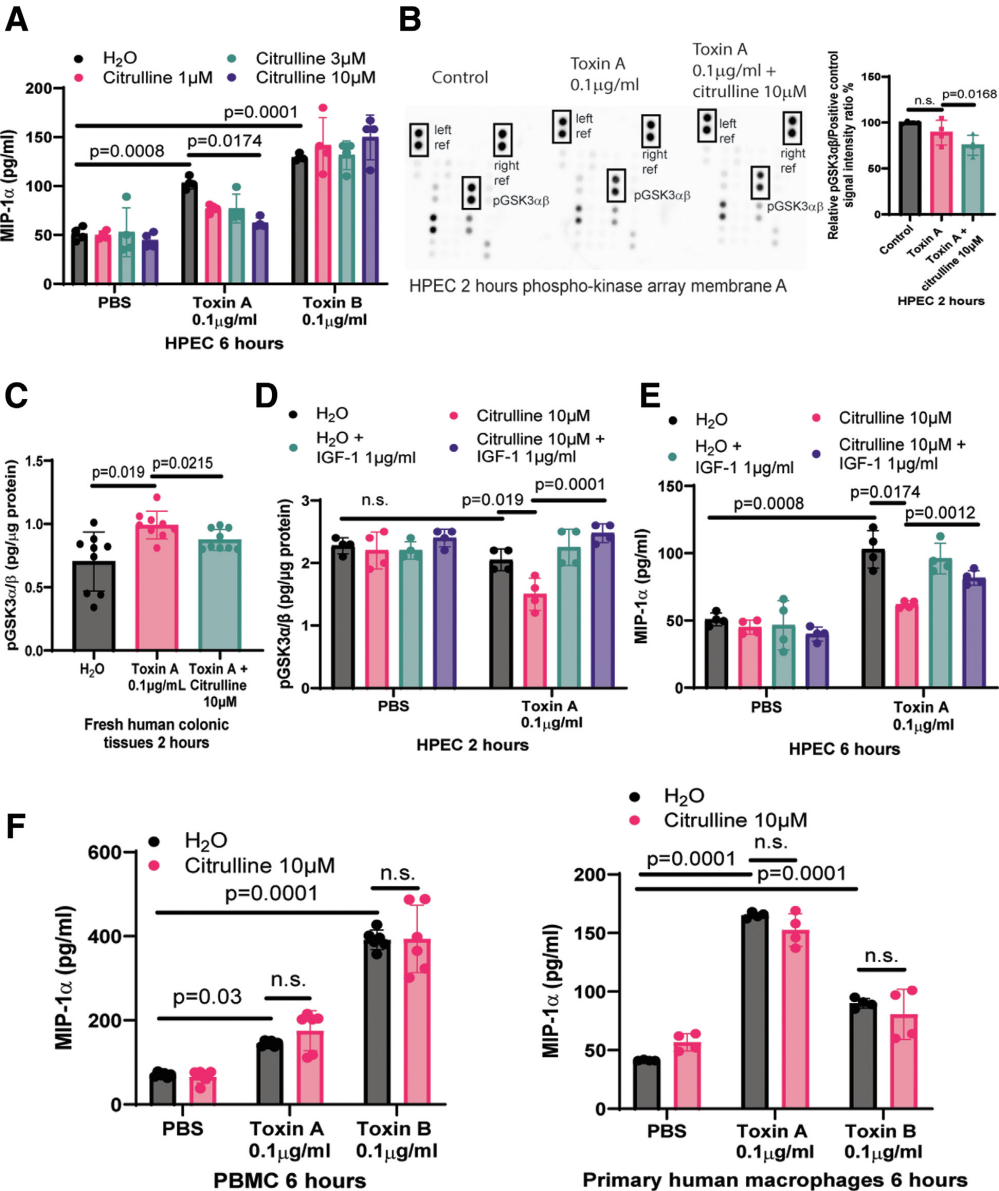
**Citrulline inhibited toxin A-mediated MIP-1α secretion with GSK3α/β dephosphorylation in HPECs.** (*A*) MIP-1α ELISA. Serum-starved HPECs were pretreated with or without citrulline for 30 minutes and then incubated with PBS, toxin A, and toxin B. MIP-1α levels in conditioned media were detected by ELISA. Results are pooled from four experiments (mean ± SD). One-way ANOVA was used. (*B*) *Left panel*: Phospho-kinase array. Serum-starved HPECs were treated with or without citrulline for 30 minutes, followed by either PBS or toxin A for 2 hours. The cells were lysed, and the lysates were assayed with the protein Proteome Profiler Human Phospho-Kinase Array Kit (ARY003C, R&D Systems). The images were captured by the Bio-Rad ChemiDoc Imaging system and analyzed by Bio-Rad Image Lab software. (*B*) *Right panel*: Relative phospho-GSK3α/β signal. Citrulline dephosphorylated GSK3α/β in toxin A-treated HPECs. Results are pooled from 4 experiments (mean ± SD). One-way ANOVA was used. (*C*) Phosphorylated GSK3α/β levels. The fresh human colonic explants were pretreated with 10 μM citrulline for 30 minutes, followed by incubation with 0.1 mg/mL toxin A for 2 hours. The phosphorylated GSK3α/β levels were determined by ELISA. Results were pooled from 4 tissue donors (mean ± SD). One-way ANOVA was used. (*D–E*) Phosphorylated GSK3α/β and MIP-1α levels. Serum-starved HPECs were pretreated with or without IGF-1 for 30 minutes, followed by citrulline for 30 minutes, and then incubated with either PBS or toxin A for either 2 or 6 hours. The citrulline-mediated GSK3α/β dephosphorylation in 2 hours and MIP-1α inhibition in 6 hours was reversed by IGF-1. Results are pooled from 4 experiments (mean ± SD). One-way ANOVA was used. (*F*) MIP-1α levels in conditioned media. PBMCs and macrophages were pretreated with citrulline for 30 minutes and then incubated with toxins for 6 hours. MIP-1α levels were measured by ELISA. Results were pooled from 6 PBMC and 4 macrophage experiments (mean ± SD). One-way ANOVA was used.

CDI patients have increased MIP-1α protein expression in colonic mucosa.^12^ Citrulline (10 μM) reduced MIP-1α secretion in toxin A-treated HPECs and fresh human colonic explants (Figures 1*D* and 14*A*). Phospho-kinase protein array and ELISA indicated citrulline-mediated GSK3α/β dephosphorylation in toxin A-treated HPECs and fresh human colonic explants (Figure 14*B–C*). Dephosphorylated GSK3αβ is active.^22^ As insulin-like growth factor I (IGF-1) can inactivate GSK3α/β,^23^ IGF-1 abolished the citrulline-mediated GSK3α/β dephosphorylation and MIP-1α inhibition in toxin A-treated HPECs (Figure 14*D–E*). On the other hand, citrulline did not affect MIP-1α secretion in toxin-treated PBMCs and macrophages (Figure 14*F*). Therefore, citrulline-dependent GSK3α/β dephosphorylation might inhibit MIP-1α secretion in toxin A-treated colonic epithelial cells.

## Discussion

This study reveals that citrulline inhibits *C. difficile*, increases IL-10 expression, and decreases MIP-1α expression in the intestine. These results, combined with our previous fecal metabolomics study,^10^ underscore the crucial role of citrulline in preventing CDI recurrence.

HSP27 modulates inflammatory responses in macrophages and protects mice from sepsis and endotoxemia.^24^, ^25^, ^26^ In patients with CDI, their low circulating HSP27 levels (by 2.78-fold) may reflect impaired responses to *C. difficile* (Table 2A, Table 2B). The citrulline-mediated HSP27 phosphorylation and IL-10 production in macrophages may be associated with the M2 polarization of macrophages toward an anti-inflammatory phenotype.^27^^,^^28^ On the other hand, the significance of citrulline-mediated HSP27 and IL-10 expression in different types of cells deserves further investigation.

In mice, neutralization of MIP-1α ameliorated CDI colitis and deficiency of CCL3 reduced toxin A-mediated enteritis.^12^^,^^29^ These findings suggested that citrulline-mediated MIP-1α inhibition might reduce toxin A-related disease activity in infected hamsters and mice (Figures 3*F* and 10*B–D*).

GSK3 phosphorylation exists in some infections.^30^ Some GSK3 inhibitors can inhibit inflammatory responses in bacterial infections.^31^ The GSK3 has regulatory roles in inflammation.^32^ Unfortunately, the relationship between citrulline, HSP27, and GSK3 in infected hamsters cannot be further explored due to the lack of hamster-specific HSP27- and GSK3-manipulating tools and assays.

NO is unnecessary to mediate citrulline’s protective mechanisms because citrulline also confers a NO-independent ergogenic benefit during exercise.^33^ Additionally, the lack of cytoprotective effect of citrulline against toxin B may be protective against CDI because intestinal epithelial cell apoptosis in CDI serves as a defensive mechanism to restrict *C. difficile* growth *in vivo*.^34^

In infected regular hamsters, 3-day oral citrulline treatment caused a 2-fold increase in cecal citrulline levels, leading to slightly lowered cecal *C. difficile* ribotype 027 abundance and toxin levels (Figures 3*C* and 4*D*). The MIC of citrulline at 44 mg/mL or 250 μM eliminated ribotype 027 (Figure 1*C*), which is much weaker than vancomycin with a MIC at <2 mg/mL or 1.38 μM.^35^^,^^36^ The susceptibility of other *C. difficile* strains to citrulline still needs additional investigations. Citrulline eliminated cecal *C. difficile* in infected ABX + HGM but not regular hamsters in 3 days because ABX + HGM hamsters had a much lower cecal *C. difficile* abundance than regular hamsters (Figures 4*D* and 8*D*). Although it is unknown how much *C. difficile* suppression is required to confer survival protection, citrulline is unlikely to inhibit CDI solely by its weak direct anti-*C. difficile* effects.

Our previous study with an anti-inflammatory drug, loratadine, demonstrated that the antibacterial effect against *C. difficile* is unnecessary for ameliorating CDI colitis.^11^ Citrulline treatment failed to reduce fecal *C. difficile* abundance and toxin levels in infected regular C57BL/6J mice (Figures 10*F* and 11*D*) because it has no antibacterial effect against *C. difficile* VPI10463.^10^ Citrulline also had no antibacterial effect on *C. difficile* ribotype 017 (Figure 1*C*). Therefore, at least for the short term, citrulline-dependent immunomodulation may help protect ribotype 017-infected HuCD34-NCG mice and VPI10463-infected regular mice.

As demonstrated by cecal microbiota transplantation, citrulline-conditioned gut microbiota conferred long-term protection against CDI recurrence in the recipient hamsters without receiving oral citrulline treatment (Figure 4*B*). In the long term, citrulline treatment might establish an unfavorable environment against *C. difficile* survival after tapering vancomycin, leading to the complete elimination of *C. difficile* on day 20 (Figures 4*D* and 8*D*). However, the influence of citrulline treatment on specific gut bacterial species is inconclusive because the relative abundance data of the cecal microbiota in hamsters and fecal microbiota in mice indicated no consistent microbiota patterns among citrulline-treated infected animals (Figures 5*C*, 6*C*, 8*C*, and 11*C*).

All antibiotic-treated hamsters had low cecal alpha diversity, especially in ABX + HGM hamsters (Figure 5, Figure 6, and 8). Citrulline increased cecal alpha diversity and altered beta diversity in vancomycin-treated regular and ABX + HGM hamsters (Figure 6*A–B* and 8*A–B*). Similarly, a study showed that citrulline supplementation increased fecal microbiota diversity and enhanced the growth performance of pigs.^37^ A diverse gut microbiota should help resist CDI recurrence.^38^

Similar to loratadine’s effects in infected mice,^11^ oral citrulline treatment increased circulating naïve CD8 T cell count in the infected HuCD34-NCG and regular mice (Figure 9*F* and Table 7). In the infected mice, the decreased proportion of circulating naïve CD8 T cells with the corresponding increased proportion of circulating memory CD8 T cells suggested immune activation, which was reversed by citrulline treatment (Table 7). Naïve CD8 T cells may help prevent CDI recurrence in mice.^39^ In citrulline-treated infected mice, the role of circulating CD8+ T-cell receptor gamma delta (TCRγΔ)-CD3+ T cells is unknown (Table 7), but we speculate that citrulline treatment may increase the availability of cytotoxic T cells to fight infection. Like loratadine,^11^ citrulline also modulated cytokine expression in colonic human CD14-expressing macrophages in HuCD34-NCG mice (Figure 9*F*). In mice, citrulline treatment should mediate its protective effect via modulating immune cell responses in circulation and colons. Unfortunately, comprehensive immunophenotyping of hamsters is currently unfeasible due to the unavailability of hamster-specific flow cytometry-compatible antibodies.

The effective dose of citrulline against CDI in mice and hamsters is 10 mg/kg/day, equivalent to 56 to 91 mg/day = (10 mg/kg × human equivalent dose: 0.08 mouse or 0.13 hamster mg/kg × 70 kg) in humans.^40^ As citrulline does not cause adverse effects to humans up to 15000 mg/day,^41^ multiple clinical trials used chronic oral citrulline supplementation at 6000 mg/day to evaluate athlete performance.^42^

There are some limitations of this study in pursuing the mechanisms of action of citrulline. For example, germ-free mice are unhelpful in studying the roles of citrulline’s anti-inflammatory effects in primary CDI without the interference of gut microbiota because all germ-free mice infected with VPI10463 developed fulminant colitis and died within 48 hours post-infection.^43^ Similarly, we used very low (100 spores per mouse) *C. difficile* to infect the germ-free mice on day 0. All positive control infected mice (*C. difficile* without citrulline) died within the first 24 hours. Even with repeated (3 doses of 10 mg/kg) citrulline treatments, all citrulline-treated infected mice still had severe diarrhea and died on day 2. Fecal sample analyses of these mice showed that citrulline failed to eliminate *C. difficile* in the intestine. We speculate that the anti-inflammatory effect should at least play some protective role in this situation. However, we could not precisely determine whether the anti-CD or anti-inflammatory effect was more important than others because it is unknown how much *C. difficile* suppression is required to confer survival.

Another limitation is that the specific role of citrulline-driven macrophage-derived IL-10 in CDI cannot be studied with macrophage-specific IL-10-deficient mice because citrulline could not induce colonic IL-10 expression in infected regular mice (Figure 10*D*, *right panel*). Similarly, no tool is available to manipulate IL-10 in macrophages in hamsters.

## Conclusion

**Figure 15:**
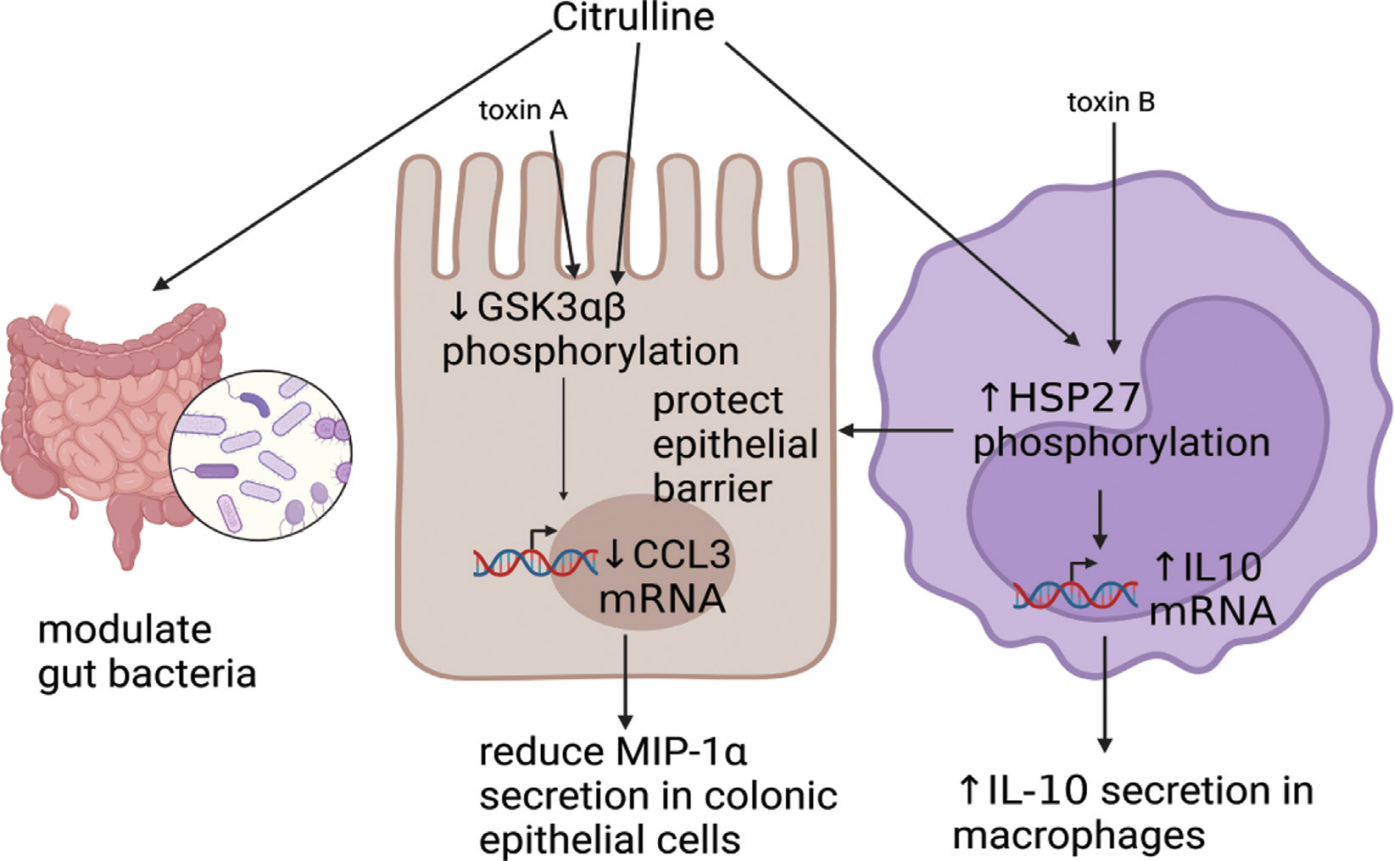
**A summary of citrulline’s effects.** The image was created by Biorender.

Citrulline prevents CDI recurrence in animal models. Its dual anti-inflammatory effects may confer protection in the intestine (Figure 15). These findings support further investigations to develop citrulline as an adjunct to antibiotic therapies to prevent CDI recurrence.

## Materials and Methods

### Chemicals

Chemical information is shown in Table 1A, Table 1B.

### Fresh Human Colonic Tissues and Sera

Fresh human colonic explants from patients with colon cancer and sera from healthy donors and patients with CDI were obtained from the University of California Los Angeles (UCLA) Pathology.^11^^,^^12^^,^^18^ The UCLA Institutional Review Board (IRB) approved this study and waived the informed consent requirement (IRB 12-001499). No patient-identifiable information was obtained. All methods were compliant with relevant guidelines and regulations. The baseline characteristics of patients are shown in Table 1A, Table 1B and Table 2A, Table 2B.

The 3 × 3 mm explants were cultured in serum-free RPMI1640 (1 mL/well) and treated with or without citrulline for 30 minutes, followed by 1 μL/mL phosphate-buffered saline (PBS), 0.1 μg/mL toxin A, and 0.1 μg/mL toxin B.^11^^,^^18^ One-half of the conditioned media was collected for the multiplex ELISA 6 hours later.^12^ Twenty-four hours later, the explants were fixed in formalin, embedded in paraffin, sectioned at 4-μm thickness, and stained with hematoxylin and eosin (H&E). Two observers blindly evaluated 2 different mucosal locations per tissue section. The severity of epithelial injury was graded on a scale of 0 to 3.^18^

Human colonic organoids were prepared as described previously.^18^

### Human Serum Cytokine and Chemokine Measurement

The sera from 10 patients per group were pooled and analyzed by RayBiotech’s human L-type array service (AAH-BLG-SERV). After normalization with control signals, the serum protein levels were converted to percentages and folds relative to healthy donors.

### HPECs and Primary Human Macrophages

HPECs (H6047, Cell Biologics) were cultured in a medium (H6621, Cell Biologics).^44^ Primary human macrophages (#70042, Stemcell Technologies) and primary human peripheral blood mononuclear cells (PBMCs) (70025, Stemcell Technologies) were cultured in RPMI1640 medium containing 10% fetal bovine serum and 1% penicillin-streptomycin. Table 1A, Table 1A shows the donors’ baseline characteristics.

After changing to serum-free media, the cells were treated with inhibitors, citrulline for 30 minutes, and *C. difficile* toxins for 2-6 hours. After 2 hours, the cells were lysed for phosphorylated heat shock protein 27 (pHSP27) ELISA (DY2314) or phosphorylated glycogen synthase kinase 3 alpha/beta (pGSK3α/β) ELISA (DYC2630) from R&D Systems. Alternatively, after 6 hours of treatment, the conditioned media were collected for MIP-1α (DY270), IL-10 (DY217B), and HSP27 (DYC1580) ELISA from R&D Systems and nitrate assay (78000100) from Cayman Chemicals.

### HTS for Anti-inflammatory Effects

The macrophages and HPECs (5000 cells/well) were pretreated with 10 μM 889 compounds from human endogenous metabolite panels (HY-L030, MedChemExpress), followed by 0.1 mg/mL *C. difficile* toxins for 6 hours, and the supernatants were collected for ELISAs.^11^

### HTS for Antibacterial Effects

Hypervirulent *C. difficile* toxin A+B+ ribotype 027 (ATCC BAA-1805) at 1 × 10^5^ spores/mL were incubated with 10 μM 889 compounds from human endogenous metabolite panels in brain heart infusion (BHI) broth with 0.1% taurocholate for 24 hours at 37 ^o^C.^11^^,^^18^ The viability of *C. difficile* was determined by absorbance at 600 nm using an Agilent Synergy HT plate reader.^10^^,^^11^^,^^18^

### Determination of Antibacterial Effects of Citrulline

Hypervirulent toxin A-B+ ribotype 017 (ATCC 43598) and *C. difficile* ribotype 027 at 1 × 10^5^ spores/mL were incubated with 0-1000 μM citrulline in BHI broth with 0.1% taurocholate for 24 hours at 37 ^o^C.^11^^,^^18^ The viability of *C. difficile* was determined by absorbance at 600 nm using an Agilent Synergy HT plate reader.^10^^,^^11^^,^^18^ The lowest concentration of citrulline that eliminated *C. difficile* in broth is MIC.

Then, the culture broth samples (100 mL/plate) were spread onto agar plates containing BHI and 0.1% taurocholate. After incubation for 24 hours at 37 ^o^C, *C. difficile* colonies on the agar plates were assessed on a lightbox. MBC is the lowest concentration of citrulline, which eliminates *C. difficile* on agar plates.

The protocol for determining the MIC and MBC of drugs for bacteria is available at https://drgermophile.com/2020/06/24/bacteriostatic-vs-bactericidal/.

### Phospho-kinase Proteome Array

Serum-starved HPECs or macrophages were treated with or without citrulline for 30 minutes, followed by PBS or *C. difficile* toxins for 2 hours. The cell lysates (300 μg protein/group) were loaded into the Proteome Profiler Human Phospho-Kinase Array Kit (ARY003C, R&D Systems).^18^ The protein array signals were detected by a Bio-Rad ChemiDoc Imaging system and analyzed by Bio-Rad Image Lab software.^18^

### Animal Experiments

Animal studies, approved by the UCLA Institutional Animal Research Committee (#2007-116) and compliant with the ARRIVE guidelines, used 8-week-old male and female C56BL/6J mice of ∼20 to 22 g body weight (#000664, Jackson Laboratories), 147- to 217-day-old female HuCD34-NCG mice of ∼22 to 25 g body weight (#695, Charles River Laboratories), and 6-week-old female Golden Syrian hamsters of ∼80 to 100 g body weight (#049, Charles River Laboratories).^10^^,^^11^^,^^18^^,^^45^

Animals were randomly assigned to cages by animal facility staff and housed at the UCLA animal facility under standard environmental conditions with a 12/12-hour light/dark period; 25 ^o^C room temperature, disposable polypropylene cages with high-efficiency particulate air (HEPA)-filtered air circulation, autoclaved white paper bedding, sterile water, and rodent chow (#7013, Envigo) *ad libitum*. All interventions were performed during the light cycle.^11^^,^^18^

### Primary CDI Models

Mice were given antibiotic-containing drinking water (from day −6 to day −3) and then switched to regular drinking water. On day −1, they were intraperitoneally injected with clindamycin (10 mg/kg), followed by inoculation with reference toxin A+B+ VPI10463 (ATCC 43255) or ribotype 017 (10^4^ spores) via oral gavage on day 0.^11^^,^^12^^,^^18^ Hamsters were given clindamycin (30 mg/kg, oral) on day −5, followed by *C. difficile* ribotype 027 inoculation (10^4^ spores) via oral gavage on day 0.^18^^,^^45^

Antibiotics-treated human gut microbiota-treated (ABX + HGM) hamsters were generated by treatment with 2 rounds of non-absorbable antibiotic cocktails (1 mg/mL of ertapenem, neomycin, and vancomycin) and a round of systemic antibiotic cocktail (1 mg/mL of ampicillin, cefoperazone, and clindamycin) in drinking water to deplete gut microbiota in the hamsters.^20^ This antibiotic regimen successfully facilitated human gut microbiota engraftment in mice. The hamsters received an intraperitoneal injection of clindamycin, followed by oral inoculation of human fecal microbiota from a healthy donor (provided by the UCLA Goodman-Luskin Microbiome Center) and *C. difficile* ribotype 027.

Some animals were treated with 10 mg/kg/day citrulline via oral gavage daily, 24 hours after *C. difficile* inoculation.

Cecal tissues were taken and homogenized in radioimmunoprecipitation assay (RIPA) buffer with protease inhibitor cocktail (#78429, ThermoFisher).^18^ After centrifugation at 2000 g for 5 minutes to remove debris, the clear supernatants of homogenates were used for determining hamster MIP-1α and IL-10 levels with ELISA (MBS033532 and MBS8819724, MyBioSource).^11^^,^^18^

Bacteria in murine fecal samples and hamster cecal contents were identified using shotgun metagenome sequencing (CosmosID), as described previously.^11^^,^^18^ Murine feces and hamster cecal contents were suspended in a cold PBS solution (100 mg/mL) and centrifuged to remove debris. The *C. difficile* toxin (ABIN1098188, antibodies-online.com) and citrulline (MBS2601236, MyBioSource) levels in the supernatants were determined by ELISA.^11^^,^^18^

### Vancomycin-dependent Relapse Models and Cecal Microbiota Transplantation

The infected mice and hamsters were treated with 20 mg/kg/day vancomycin from day 1 to day 5 and citrulline (10 mg/kg/day) from day 1 to day 10 via oral gavage.^11^^,^^18^ The animals were monitored until day 20.

Cecal contents from vancomycin-treated and citrulline-treated donor hamsters on day 5 post-infection were collected and homogenized in ice-cold PBS (1 g/mL), followed by centrifugation. The supernatant of cecal material from one donor hamster was transferred to one vancomycin-treated recipient hamster on day 5 to 8 post-infection via oral gavage (100 mL/hamster).^46^

### Histologic Evaluation

Cecal and colonic tissues were fixed in formalin, paraffin-embedded, sectioned (4 mm), and stained with H&E.^11^^,^^18^ Two observers evaluated 3 different locations per tissue section. Colitis and cecitis severity were graded using 3 parameters: (1) epithelial tissue damage; (2) hemorrhagic congestion and mucosal edema; and (3) neutrophil infiltration. Each parameter was assigned a score of 0 to 3.^47^ The histology score was a sum of the 3 parameters (0–9).

### Multiplex ELISA

Human 27-plex multiplex ELISA (#m500kcaf0y, Bio-Rad) kits were used per the manufacturer’s instructions. Mixtures of 25 μL undiluted samples and 25 μL magnetic beads were incubated overnight at 4 °C while shaking. After washing the plates twice with wash buffer in a Biotek ELx405 washer, 25 μL of biotinylated detection antibody was added and incubated for 1 hour at room temperature. Twenty-five μL streptavidin-phycoerythrin conjugate was added to the reaction mixture and incubated for 30 minutes. Following 2 washes, beads were resuspended in sheath fluid, and fluorescence was quantified using a Luminex 200TM instrument.^12^

### Real-time reverse transcription-polymerase chain reaction

Total RNA was isolated (RNeasy, #74104, Qiagen) and reverse transcribed into cDNA (high-capacity cDNA RT kit. #4368813, ThermoFisher). PCR reactions were conducted using the Fast Universal PCR master mix (#4352042, ThermoFisher) and cataloged assays (ThermoFisher) in a Bio-Rad CFX384 system. After normalization with human 18S rRNA and mouse Gapdh, relative mRNA quantification was performed by comparing the test vs control groups. The fold changes are expressed as 2ΔΔCt. Fold-change values greater than 1 indicate a positive- or an up-regulation, and the fold-regulation equals the fold-change. Conversely, fold-change values less than 1 indicate a negative- or down-regulation, and the fold-regulation is the negative inverse of the fold-change.^11^^,^^18^

### Immunofluorescence Staining

Paraffin was removed from the tissue sections with xylene, followed by rehydration with graded ethanol. Peroxidase activity was blocked with 3% H_2_O_2_ in methanol for 10 minutes. Next, heat-induced antigen retrieval (HIER) was carried out in 0.01 M citrate buffer, pH = 6, using a Biocare decloaker at 95 ^o^C for 25 minutes. After treatment with blocking buffer for 1 hour, the sections were incubated overnight at 4 ^o^C with primary antibodies to EGF-like module-containing mucin-like hormone receptor-like 1 (EMR1) (ab254293, Abcam) and IL-10 (ab217941, Abcam) in 2% bovine serum albumin (BSA) at 1:100 dilution. On day 2, the sections were incubated with an anti-rabbit secondary antibody and 4′,6-diamidino-2-phenylindole (DAPI) (blue) nuclear stain. These multiplex reagents were included in the OPAL staining kit. The tissue sections were scanned by the Leica Aperio Versa system and analyzed using Phenochart 1.1 and Adobe Photoshop.^18^^,^^48^ The red EMR1 and yellow/green IL-10 signals were normalized against the blue nuclear signal, and then the IL-10/EMR1 ratios were calculated.

### Statistical Analysis

Unpaired Student *t*-tests were utilized for 2-group comparisons of continuous data. Two-way analyses of variance (ANOVAs) were used for multiple-group comparisons (Prism 10). Results were expressed as mean ± standard deviation (SD). Significant *P* values are shown in each figure.

## References

[1] Cornely O.A., Nathwani D., Ivanescu C. (2014). Clinical efficacy of fidaxomicin compared with vancomycin and metronidazole in Clostridium difficile infections: a meta-analysis and indirect treatment comparison. J Antimicrob Chemother.

[2] Eyre D.W., Walker A.S., Wyllie D. (2012). Infections in Oxfordshire Research Database. Predictors of first recurrence of Clostridium difficile infection: implications for initial management. Clin Infect Dis.

[3] Louie T.J., Miller M.A., Mullane K.M., OPT-80-003 Clinical Study Group (2011). Fidaxomicin versus vancomycin for Clostridium difficile infection. N Engl J Med.

[4] Bartsch S.M., Umscheid C.A., Fishman N., Lee B.Y. (2013). Is fidaxomicin worth the cost? An economic analysis. Clin Infect Dis.

[5] Waqas M., Mohib K., Saleem A. (2022). Ritelimin therapy for patients with metronidazole-unresponsive Clostridium difficile infection. Cureus.

[6] Wilcox M.H., Gerding D.N., Poxton I.R., MODIFY I and MODIFY II Investigators (2017). Bezlotoxumab for prevention of recurrent Clostridium difficile infection. N Engl J Med.

[7] Singh T., Bedi P., Bumrah K. (2019). Updates in treatment of recurrent Clostridium difficile infection. J Clin Med Res.

[8] Khoruts A., Staley C., Sadowsky M.J. (2021). Faecal microbiota transplantation for Clostridioides difficile: mechanisms and pharmacology. Nat Rev Gastroenterol Hepatol.

[9] Feuerstadt P., Aroniadis O.C., Svedlund F.L. (2022). Heterogeneity of randomized controlled trials of fecal microbiota transplantation in recurrent Clostridioides difficile infection. Dig Dis Sci.

[10] Wang J., Ghali S., Xu C. (2018). Ceragenin CSA13 reduces Clostridium difficile infection in mice by modulating the intestinal microbiome and metabolites. Gastroenterology.

[11] Xie Y., Irwin S., Chupina Estrada A. (2024). Loratadine is an anti-inflammatory agent against C. difficile toxin B. J Infect Dis.

[12] Wang J., Ortiz C., Fontenot L. (2020). The therapeutic mechanism macrophage inflammatory protein 1 alpha (MIP-1alpha/CCL3) neutralizing antibody in Clostridium difficile infection in mice. J Infect Dis.

[13] Lee S.H., Starkey P.M., Gordon S. (1985). Quantitative analysis of total macrophage content in adult mouse tissues. Immunochemical studies with monoclonal antibody F4/80. J Exp Med.

[14] Morhardt T.L., Hayashi A., Ochi T. (2019). IL-10 produced by macrophages regulates epithelial integrity in the small intestine. Sci Rep.

[15] Breuillard C., Bonhomme S., Couderc R. (2015). In vitro anti-inflammatory effects of citrulline on peritoneal macrophages in Zucker diabetic fatty rats. Br J Nutr.

[16] Yin L., Wei X., Zhang Y. (2023). Citrulline inhibits LPS-induced pyroptosis of RAW264.7 macrophages through NF-kappaB signaling pathway. Immun Inflamm Dis.

[17] Yamagishi Y., Someya A., Nagaoka I. (2020). Citrulline cooperatively exerts an anti-inflammatory effect on synovial cells with glucosamine and N-acetylglucosamine. Biomed Rep.

[18] Xie Y., Fontenot L., Chupina Estrada A. (2023). Genistein inhibits C. difficile infection via estrogen receptors and lysine deficient protein kinase 1. J Infect Dis.

[19] Fatima R., Aziz M. (2019). The hypervirulent strain of Clostridium difficile: NAP1/B1/027 - a brief overview. Cureus.

[20] Staley C., Kaiser T., Beura L.K. (2017). Stable engraftment of human microbiota into mice with a single oral gavage following antibiotic conditioning. Microbiome.

[21] Zhang Y., Yang Z., Gao S. (2017). The role of purified Clostridium difficile glucosylating toxins in disease pathogenesis utilizing a murine cecum injection model. Anaerobe.

[22] Beurel E., Grieco S.F., Jope R.S. (2015). Glycogen synthase kinase-3 (GSK3): regulation, actions, and diseases. Pharmacol Ther.

[23] Fang X., Yu S.X., Lu Y. (2000). Phosphorylation and inactivation of glycogen synthase kinase 3 by protein kinase A. Proc Natl Acad Sci U S A.

[24] Breed E.R., Hilliard C.A., Yoseph B. (2018). The small heat shock protein HSPB1 protects mice from sepsis. Sci Rep.

[25] You W., Min X., Zhang X. (2009). Cardiac-specific expression of heat shock protein 27 attenuated endotoxin-induced cardiac dysfunction and mortality in mice through a PI3K/Akt-dependent mechanism. Shock.

[26] Salari S., Seibert T., Chen Y.X. (2013). Extracellular HSP27 acts as a signaling molecule to activate NF-kappaB in macrophages. Cell Stress Chaperones.

[27] Bogel G., Muranyi J., Szokol B. (2022). Production of NOS2 and inflammatory cytokines is reduced by selected protein kinase inhibitors with partial repolarization of HL-60 derived and human blood macrophages. Heliyon.

[28] Chuang Y., Hung M.E., Cangelose B.K., Leonard J.N. (2016). Regulation of the IL-10-driven macrophage phenotype under incoherent stimuli. Innate Immun.

[29] Morteau O., Castagliuolo I., Mykoniatis A. (2002). Genetic deficiency in the chemokine receptor CCR1 protects against acute Clostridium difficile toxin A enteritis in mice. Gastroenterology.

[30] Hoffmeister L., Diekmann M., Brand K., Huber R. (2020). GSK3: a kinase balancing promotion and resolution of inflammation. Cells.

[31] Wang H., Kumar A., Lamont R.J., Scott D.A. (2014). GSK3beta and the control of infectious bacterial diseases. Trends Microbiol.

[32] Jope R.S., Yuskaitis C.J., Beurel E. (2007). Glycogen synthase kinase-3 (GSK3): inflammation, diseases, and therapeutics. Neurochem Res.

[33] Gough L.A., Sparks S.A., McNaughton L.R. (2021). A critical review of citrulline malate supplementation and exercise performance. Eur J Appl Physiol.

[34] Saavedra P.H.V., Huang L., Ghazavi F. (2018). Apoptosis of intestinal epithelial cells restricts Clostridium difficile infection in a model of pseudomembranous colitis. Nat Commun.

[35] Buchler A.C., Rampini S.K., Stelling S. (2014). Antibiotic susceptibility of Clostridium difficile is similar worldwide over two decades despite widespread use of broad-spectrum antibiotics: an analysis done at the University Hospital of Zurich. BMC Infect Dis.

[36] Aspevall O., Lundberg A., Burman L.G. (2006). Antimicrobial susceptibility pattern of Clostridium difficile and its relation to PCR ribotypes in a Swedish university hospital. Antimicrob Agents Chemother.

[37] Du J., Gan M., Xie Z. (2023). Effects of dietary L-Citrulline supplementation on growth performance, meat quality, and fecal microbial composition in finishing pigs. Front Microbiol.

[38] Seekatz A.M., Young V.B. (2014). Clostridium difficile and the microbiota. J Clin Invest.

[39] Mileto S.J., Hutton M.L., Walton S.L. (2022). Bezlotoxumab prevents extraintestinal organ damage induced by Clostridioides difficile infection. Gut Microbes.

[40] Nair A.B., Jacob S. (2016). A simple practice guide for dose conversion between animals and human. J Basic Clin Pharm.

[41] Moinard C., Nicolis I., Neveux N. (2008). Dose-ranging effects of citrulline administration on plasma amino acids and hormonal patterns in healthy subjects: the Citrudose pharmacokinetic study. Br J Nutr.

[42] Figueroa A., Wong A., Jaime S.J., Gonzales J.U. (2017). Influence of L-citrulline and watermelon supplementation on vascular function and exercise performance. Curr Opin Clin Nutr Metab Care.

[43] Reeves A.E., Koenigsknecht M.J., Bergin I.L., Young V.B. (2012). Suppression of Clostridium difficile in the gastrointestinal tracts of germfree mice inoculated with a murine isolate from the family Lachnospiraceae. Infect Immun.

[44] Xie Y., Fontenot L., Estrada A.C. (2022). Elafin reverses intestinal fibrosis by inhibiting cathepsin S-mediated protease-activated receptor 2. Cell Mol Gastroenterol Hepatol.

[45] Koon H.W., Su B., Xu C. (2016). Probiotic Saccharomyces boulardii CNCM I-745 prevents outbreak-associated Clostridium difficile-associated cecal inflammation in hamsters. Am J Physiol Gastrointest Liver Physiol.

[46] Wang J., Ortiz C., Fontenot L. (2020). Elafin inhibits obesity, hyperglycemia, and liver steatosis in high-fat diet-treated male mice. Sci Rep.

[47] Pothoulakis C., Castagliuolo I., LaMont J.T. (1994). CP-96,345, a substance P antagonist, inhibits rat intestinal responses to Clostridium difficile toxin A but not cholera toxin. Proc Natl Acad Sci U S A.

[48] Xie Y., Chupina Estrada A., Nelson B. (2022). ADS024, a Bacillus velezensis strain, protects human colonic epithelial cells against C. difficile toxin-mediated apoptosis. Front Microbiol.

